# Effective clearance of rituximab-resistant tumor cells by breaking the mirror-symmetry of immunoglobulin G and simultaneous binding to CD55 and CD20

**DOI:** 10.1038/s41598-023-45491-8

**Published:** 2023-10-25

**Authors:** Sang Min Lee, Sung-Won Min, Hyeong Sun Kwon, Gong-Deuk Bae, Ji Hae Jung, Hye In Park, Seung Hyeon Lee, Chung Su Lim, Byoung Joon Ko, Ji Chul Lee, Sang Taek Jung

**Affiliations:** 1 Department of Biomedical Sciences, Graduate School of Medicine, Korea University, 73 Goryeodae-ro, Seongbuk-gu, Seoul, 02841 Republic of Korea; 2 Department of Applied Chemistry, Kookmin University, 77, Jeongneung-ro, Seongbuk-gu, Seoul, 02707 Republic of Korea; 3 SG Medical, 3-11, Ogeum-ro 13-gil, Songpa-gu, Seoul, 05548 Republic of Korea; 4 New Drug Development Center, Osong Medical Innovation Foundation 123, Cheongju, Chungcheongbuk-do 28160 Republic of Korea; 5 School of Biopharmaceutical and Medical Science, Sungshin Women’s University, 55, Dobonng-Ro 76ga-gil, Gangbuk, Seoul, 01133 Republic of Korea; 6 BK21 Graduate Program, Department of Biomedical Sciences, Korea University College of Medicine, Seoul, Republic of Korea; 7 Institute of Human Genetics, Korea University College of Medicine, Seoul, 02841 Republic of Korea; 8 Biomedical Research Center, Korea University Anam Hospital, Seoul, 02841 Republic of Korea

**Keywords:** Complement, Targeted therapies, Cancer therapeutic resistance

## Abstract

Complement-dependent cytotoxicity (CDC), which eliminates aberrant target cells through the assembly and complex formation of serum complement molecules, is one of the major effector functions of anticancer therapeutic antibodies*.* In this study, we discovered that breaking the symmetry of natural immunoglobulin G (IgG) antibodies significantly increased the CDC activity of anti-CD20 antibodies. In addition, the expression of CD55 (a checkpoint inhibitor in the CDC cascade) was significantly increased in a rituximab-resistant cell line generated in-house, suggesting that CD55 overexpression might be a mechanism by which cancer cells acquire rituximab resistance. Based on these findings, we developed an asymmetric bispecific antibody (SBU-CD55 × CD20) that simultaneously targets both CD55 and CD20 to effectively eliminate rituximab-resistant cancer cells. In various cancer cell lines, including rituximab-resistant lymphoma cells, the SBU-CD55 × CD20 antibody showed significantly higher CDC activity than either anti-CD20 IgG antibody alone or a combination of anti-CD20 IgG antibody and anti-CD55 IgG antibody. Furthermore, the asymmetric bispecific antibody (SBU-CD55 × CD20) exhibited significantly higher CDC activity against rituximab-resistant cancer cells compared to other bispecific antibodies with symmetric features. These results demonstrate that enhancing CDC with an asymmetric CD55-binding bispecific antibody could be a new strategy for developing therapeutics to treat patients with relapsed or refractory cancers.

## Introduction

A natural IgG molecule in the human immune system has a mirror-symmetric structure^1,2^ and binds to its target antigen in a bivalent form, with extremely high affinity and specificity. The exceptional superiority of IgG antibody molecules as binding agents compared to small molecules has enabled the use of monoclonal antibodies in the treatment of various types of cancer^3^. As of October 24, 2022, 140 therapeutic antibodies have been approved by the US Food and Drug Administration (FDA) and the European Medicines Agency (EMA). Of these, 64 antibodies (46%) have been used to treat cancer^4,5^. Furthermore, various antibody engineering technologies that have accumulated over the past few decades have enabled the development of therapeutic bispecific antibodies with novel functions that can overcome the limitations of natural monoclonal IgG antibodies that are specific to a single epitope^6^. Seven bispecific antibodies have been approved by the FDA or EMA, and more than 100 are in clinical trials^5,7,8^. The market for bispecific anticancer antibodies is expected to reach US$3.7 billion by 2027^5,9^.

After the Fab (Fragment antigen binding) region of an IgG antibody recognizes and binds to an antigen, the antibody fragment crystallizable (Fc) region activates various immune leukocytes through interactions with FcγRs (Fcgamma receptors) that induce antibody-dependent cell-mediated cytotoxicity and antibody-dependent cell-mediated phagocytosis to eliminate target cancer cells. In addition, the Fc–C1q interaction initiates complement-dependent cytotoxicity (CDC) and generates membrane attack complexes through the assembly of various serum complement molecules, leading to tumor cell lysis^10–12^. Among the effector functions, CDC plays a key role in the efficacy of a number of anticancer antibodies, including rituximab (Rituxan®; anti-CD20), the first anticancer antibody approved by the US FDA^13,14^.

To improve CDC, many efforts have been made to increase the binding affinity between the Fc region and serum complement C1q by using an amino acid substitution or glycan modification in the antibody Fc region^15–17^. In addition, since the binding structure between the hexameric head of C1q and the IgG hexamer has been identified at the molecular level^18,19^, Fc engineering strategies using these molecular insights have been attempted to improve CDC^18,20^. CDC is influenced not only by the structural characteristics of an antibody, but also by the type, characteristics, expression level, and epitope location of the antigen^18,21,22^.

Tumor cells express membrane-bound complement regulatory proteins (mCRPs; CD35, CD46, CD55, and CD59) that normal cells use to prevent excessive activation of complement cascades in the early stages of an immune response^23^. Thus, tumor cells evade the complement-mediated target cell clearance mechanism of IgG antibodies for survival by hijacking the complement regulatory mechanism^24^. Among mCRPs, CD55 (decay-accelerating factor (DAF)) accelerates the degradation of C3 convertase and has been reported to be overexpressed on the cell surface of various cancers, particularly breast cancer^25^, leukemia^26^, colorectal cancer^27^, and gastric cancer^28^. Therefore, a strategy to block CD55 has been proposed to overcome the limitations of therapeutic anticancer antibodies^29,30^.

In this study, we hypothesized that altering the mirror symmetry of the Fab arms in human IgG molecules could influence the arrangement of opsonized antibodies on the target cell surface and potentially induce variations in hexamerization, essential for initiating C1q binding and CDC effector function. Unexpectedly, our asymmetric variant of the anti-CD20 antibody markedly outperformed the clinical-grade rituximab with a symmetric structure in terms of enhanced CDC efficacy. To build upon this finding, we crafted SBU-CD55 × CD20, an asymmetric bispecific antibody uniquely designed to simultaneously target CD20 and the complement regulatory protein CD55. Notably, this innovative antibody excelled in promoting CDC-mediated tumor cell elimination, surpassing the effectiveness of both rituximab and a combined rituximab/anti-CD55 antibody (4-1H). Intriguingly, such marked improvement in CDC was absent in symmetric bispecific antibodies. Our results suggest that an asymmetric bispecific antibody that simultaneously targets CD55 and a tumor-specific antigen could improve tumor cell-killing activity beyond that demonstrated by conventional symmetric monospecific or bispecific antibodies.

## Results

### Design and production of an asymmetric anti-CD20 antibody (SBU-CD20)

**Figure 1:**
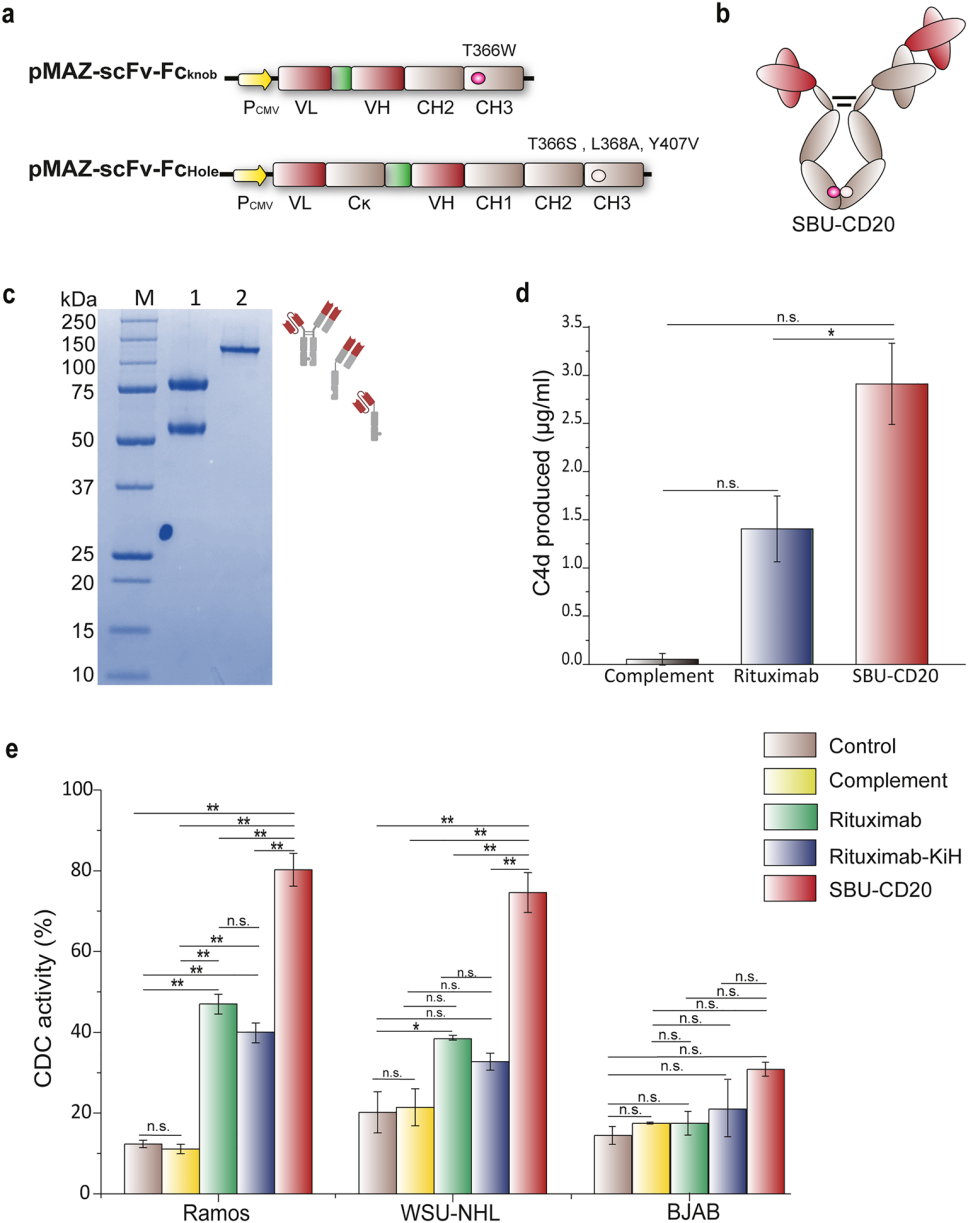
Production of an asymmetric anti-CD20 antibody (SBU-CD20: scFv-Fc_knob_—scFab-Fc_Hole_) and analysis of its CDC. (**a**) Expression cassette for SBU-CD20. Pink and white circles indicate a knob mutation (T366W) and hole mutations (T366S, L368A, and Y407), respectively. (**b**) Schematic showing the structure of SBU-CD20. (**c**) SDS-PAGE showing purified SBU-CD20, Lane 1: reduced SBU-CD20; Lane 2: nonreduced SBU-CD20. (**d**) C4d production of SBU-CD20 and rituximab. Error bars represent the SE (standard error) of three replicates. (**e**) CDC of rituximab, rituximab-KiH, and SBU-CD20 in rituximab-sensitive cell lines (Ramos and WSU-NHL) and a rituximab-resistant cell line (BJAB). Error bars represent standard error calculated from triplicate samples. *P*-values < 0.05, < 0.01, and > 0.05 are marked with *, **, and “n.s.” for not significant, respectively.

The CDC of an antibody is influenced by the isotype and structural features of the IgG molecule^21,22^. To investigate how breaking the mirror symmetry of an IgG antibody alters its CDC for tumor cell clearance, we designed an asymmetric heterodimeric antibody that we named the Specific Bifunctional Unit (SBU). For efficient heterodimerization, CDC effector function, and prolonged circulating half-life, the SBU (scFv-Fc_Knob_–scFab-Fc_Hole_) consists of an Fc region of human IgG1 with a Knob mutation (T366W) and hole mutations (T366S, L368A, and Y407)^31^ in each Fc polypeptide. In addition, the antigen-binding region was composed of a single-chain variable fragment (scFv) and a single-chain antigen binding fragment (scFab) to remove side products, such as Knob-Knob homodimers (scFv-Fc_Knob_–scFv-Fc_Knob_) and Knob monomers (scFv–Fc_Knob_) using the IgG Ck region-binding KappaSelect resin^32,33^, while preventing mispairing between the H-chain and L-chain of the antibody (Fig. 1a). We designed two symmetric anti-CD20 IgG antibodies, a commercial anti-CD20 IgG antibody (rituximab: Rituxan®), and an IgG antibody (rituximab-KiH) containing a Knob into Hole mutations, respectively. Additionally, we designed SBU-CD20, an asymmetric anti-CD20 antibody that is an asymmetric form of rituximab and contains two variable domains, VH and VL sequences derived from rituximab.

After expressing SBU-CD20 (scFv-Fc_Knob_–scFab-Fc_Hole_) in Expi293F cells and performing a simple one, step affinity chromatography using KappaSelect resin, we obtained highly purified SBU-CD20 (Fig. 1b). SEC and RP-HPLC analyses showed that the purity of SBU-CD20 was similar to that of symmetric anti-CD20 IgG antibodies, rituximab and rituximab-KiH, which were prepared using the same steps (100% and 99.85% for rituximab, 100% and 99.86% for rituximab-KiH, and 98.92% and 97.18% for SBU-CD20 in the SEC and RP-HPLC analyses, respectively) (Supplementary Fig. S2). Interestingly, no band corresponding to undesired monomeric scFab–Fc_Hole_ was detected in the SBU-CD20 sample purified with KappaSelect resin (Fig. 1c); Supplementary Fig. S1a), suggesting that almost all of the expressed monomeric scFab–Fc_Hole_ molecules might have assembled into heterodimers with scFv–Fc_Knob_ to form SBU-CD20 because of the low homodimerization tendency of scFab–Fc_Hole_, which might have a slightly lower expression level than scFv–Fc_Knob_. In contrast, when SBU-CD20 was purified using the Fc region–binding Protein A resin instead of the KappaSelect resin, unwanted by-products such as Knob-Knob homodimers (scFv-Fc_Knob_–scFv-Fc_Knob_) and Knob monomers (scFv–Fc_Knob_) were detected in the eluent (Supplementary Fig. S1a), and the purity of the SBU-CD20 decreased to 84% in the RP-HPLC analysis (Supplementary Fig. S1b).

### SBU-CD20 with broken mirror-symmetry elicits higher CDC activity than anti-CD20 antibodies with symmetric Fab arms (rituximab or rituximab-KiH)

Before comparing the CDC activities of the three anti-CD20 antibodies (SBU-CD20, rituximab, and rituximab-KiH), we analyzed their binding to the antigen and C1q. Since rituximab is known to bind to the large loop region (^168–^EPANPSEK^–175^) of CD20 on the surface of B cells^34^, we fused a peptide from amino acids 163 to the 187 to streptavidin and prepared mammalian cells (Supplementary Fig. S3). In ELISA analysis using the purified streptavidin-fused CD20 epitope peptide, SBU-CD20 showed a binding affinity similar to that of rituximab and rituximab-KiH (Supplementary Fig. S4a). In addition, SBU-CD20 showed C1q binding affinity that was almost identical to that of rituximab and rituximab-KiH (Supplementary Fig. S4b), indicating that the Knob-into-Hole mutations (T366W/T366S, L368A, and Y407) introduced into Fc for heterodimer formation and the linkers used in scFv/scFab had a negligible effect on binding to the antigen (CD20) and C1q.

On the other hand, SBU-CD20 showed approximately three times higher production of C4d molecules (intermediate products in the complement activation cascade) than rituximab (3.2 μg/ml vs. 1.1 μg/ml) (Fig. 1d). Next, we investigated the CDC of the prepared anti-CD20 antibodies using three lymphoma B-cell lines (Ramos, WSU-NHL, and BJAB). As shown in Fig. 1e, treatment with human complement sera alone resulted in 11–21% lysis of lymphoma B cells, and samples treated with human complement sera and rituximab at a concentration of 20 μg/ml resulted in exhibited 47%, 38%, and 16% lysis of Ramos, WSU-NHL, and BJAB cells, respectively. These results are in agreement with the report by Golay et al.^35^, which indicated that BJAB cells were rituximab-resistant to complement-mediated lysis. When treated with rituximab-KiH under the same conditions, the activity of CDC against the three CD20-expressing target cell lines (Ramos, WSU-NHL, and BJAB) was almost identical to that of rituximab (40%, 32%, and 20%, respectively). In sharp contrast, SBU-CD20 with an asymmetric structure exhibited CDC of 80%, 75%, and 30% against, Ramos, WSU-NHL, and BJAB, respectively, indicating dramatically improved tumor cell clearance activity.

### Increased CD55 expression on the surfaces of rituximab-resistant cells

**Figure 2:**
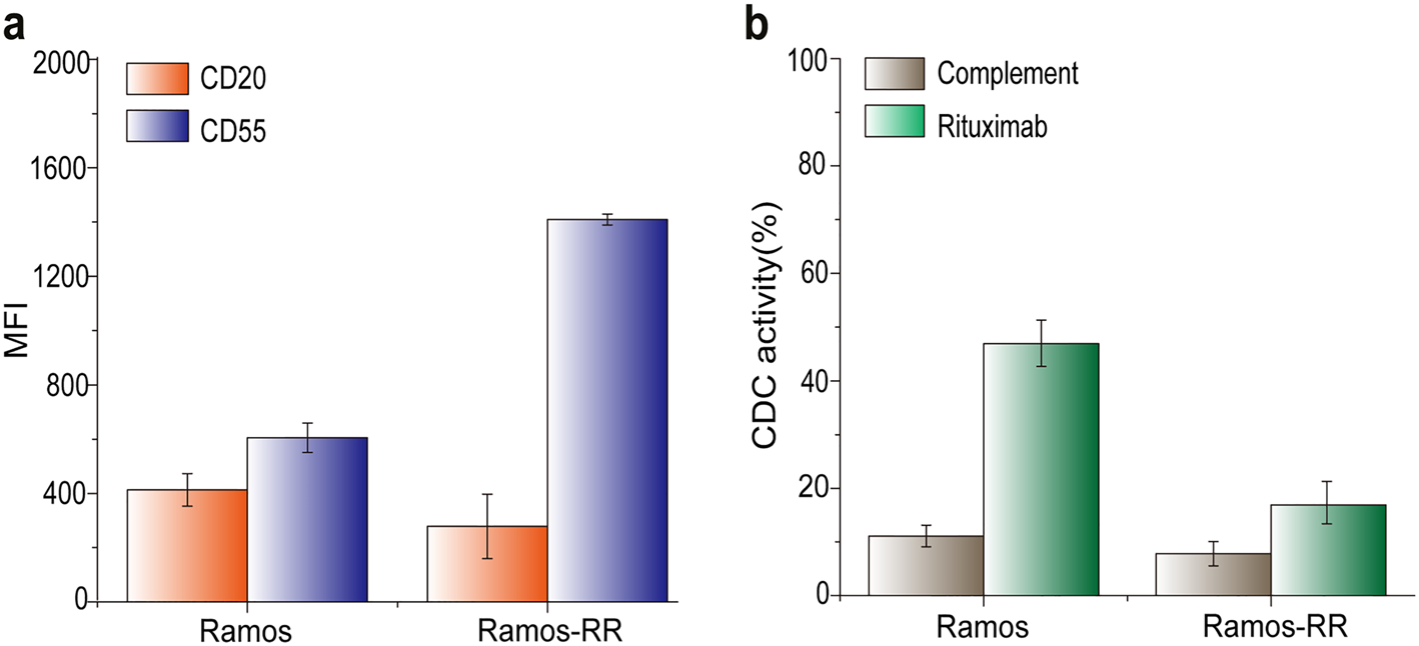
Changes in CD20/CD55 expression and CDC upon the acquisition of rituximab-resistance. (**a**) Expression of CD20 and CD55 in a rituximab-sensitive cell line (Ramos) and a rituximab-resistant cell line (Ramos-RR). Mean fluorescence intensity (MFI) upon binding to anti-human IgG antibody-FITC was measured by a FACS analysis. (**b**) CDC in the Ramos and Ramos-RR cell lines treated with rituximab. Error bars represent the SE (standard error) of three independent experiments.

Motivated by the results that (i) antibodies with an asymmetric structure could have dramatically increased tumor cell killing effects via CDC effector function and (ii) the heterodimeric SBU-CD20 antibody could be efficiently produced by simple expression and purification, we attempted to improve the low tumor cell-killing potency of existing anticancer antibodies by developing an asymmetric bispecific antibody with new antigen-binding capability and increased CDC that bypasses tumor resistance. First, to find an effective target antigen to combine with CD20 for the clearance of rituximab-resistant tumors, we constructed in-house rituximab-resistant cells (Ramos-RR) by continuously treating a rituximab-sensitive cell line (Ramos) with rituximab. As expected, rituximab treatment in the presence of complements induced cell lysis in 46.98% of Ramos cells but only 16.91% of Ramos-RR cells (Fig. 2). Next, we focused on CD55, a membrane-bound complement regulatory protein, in tumor cells that inhibits CDC (Supplementary Fig. S5). We analyzed the changes in CD55 and CD20 expression resulting from the acquisition of rituximab resistance by examining Ramos and Ramos-RR cells. When the expression levels of CD20 and CD55 on the surfaces of tumor cells were analyzed by FACS using anti-CD20 IgG antibody (rituximab) and anti-CD55 IgG antibody (4-1H)^36^, we found that CD20 expression was significantly lower in Ramos-RR cells (MFI_Ramos, CD20_ = 413.5; MFI_Ramos-RR, CD20_ = 278.0). In contrast, CD55 expression was significantly increased in rituximab-resistant cells (MFI_Ramos, CD55_ = 605.5; MFI_Ramos-RR, CD55_ = 1 409.5). These results indicate that CD55 overexpression is highly correlated with rituximab resistance, suggesting that the CDC of anti-CD20 antibodies could be improved by simultaneously targeting CD55.

### SBU-CD55 × CD20 exhibits excellent physicochemical properties and developability

**Figure 3:**
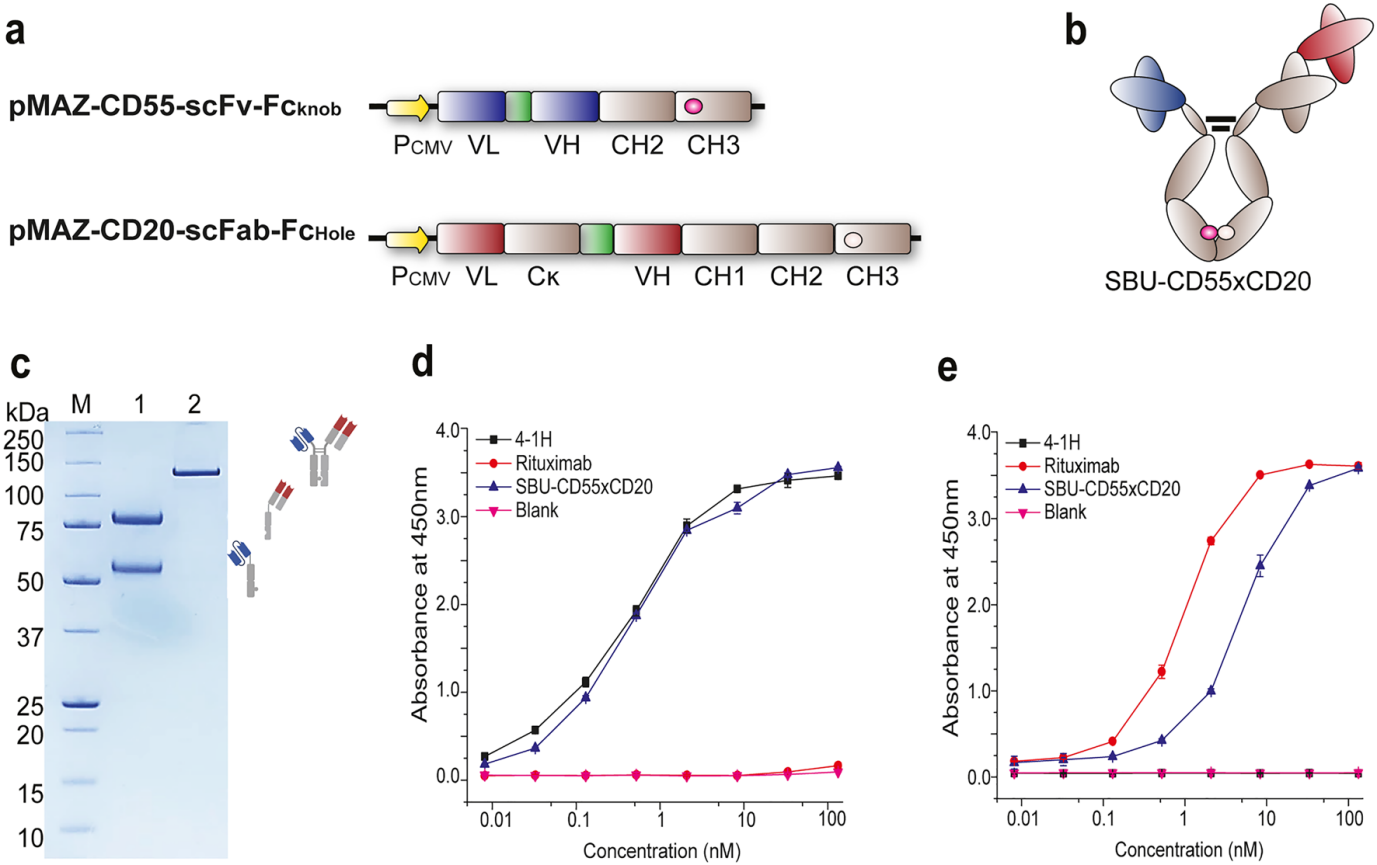
Expression, purification, and antigen binding characteristics of SBU-CD55 × CD20. (**a**) Expression cassette for SBU-CD55 × CD20. Pink and white circles indicate a knob mutation (T366W) and hole mutations (T366S, L368A, and Y407), respectively. (**b**) Schematic illustration of the structure of SBU-CD55xCD20. (**c**) SDS-PAGE showing the purified SBU-CD55 × CD20, Lane 1: reduced SBU-CD55 × CD20; Lane 2: nonreduced SBU-CD55 × CD20. (**d**,**e**) ELISA to show the binding of SBU-CD55 × CD20 to CD55 (**d**) and CD20 (**e**). Antigen binding ELISA signals for SBU-CD20 were compared with those for monoclonal IgG antibodies (rituximab and 4-1H). Error bars indicate the standard error (SE) from duplicate runs of the same sample.

In our previous study, a chimeric anti-CD55 (4-1H) monoclonal antibody with high binding affinity to CD55 (EC_50_: 0.22 nM) was identified using a chicken phage-display scFv library. The radioisotope-labeled anti-CD55 antibody conjugated with ^177^Lu demonstrated its potential as a therapeutic agent by significantly reducing tumor growth and increasing the median survival time in a pleural metastatic lung cancer mouse model^37^. To confirm that the anti-CD55 antibody (4-1H) could be effectively incorporated into our asymmetric bispecific antibody (SBU) for simultaneous targeting of both CD55 and a tumor-associated antigen (e.g. CD20, HER2, or EGFR), the expression plasmid for CD55-scFv-Fc_Knob_ was co-transfected into Expi293F cells with a plasmid encoding single-chain Fabs (CD20-scFab-Fc_Hole_, HER2-scFab-Fc_Hole_, or EGFR-scFab-Fc_Hole_ derived from the variable regions of rituximab, trastuzumab, or cetuximab, respectively) (Fig. 3a,b). Using the same expression and purification steps described for the production of the SBU-CD20 antibody, we obtained highly purified asymmetric bispecific antibodies (SBU-CD55 × CD20, SBU-CD55 × HER2, and SBU-CD55 × EGFR) that simultaneously bound two different antigens (Fig. 3c; Supplementary Fig. S6a–e). In particular, SBU-CD55 × CD20, which has a monovalent CD55 binding site, showed an apparent binding affinity similar to that of the 4-1H IgG antibody with bivalent CD55-binding paratopes (Fig. 3d). On the other hand, SBU-CD55 × CD20, which has a monovalent CD20 binding site showed a slightly lower apparent CD20 antigen binding ELISA signal than rituximab, which has bivalent antigen-binding capability, due to decreased avidity (Fig. 3e).

**Figure 4:**
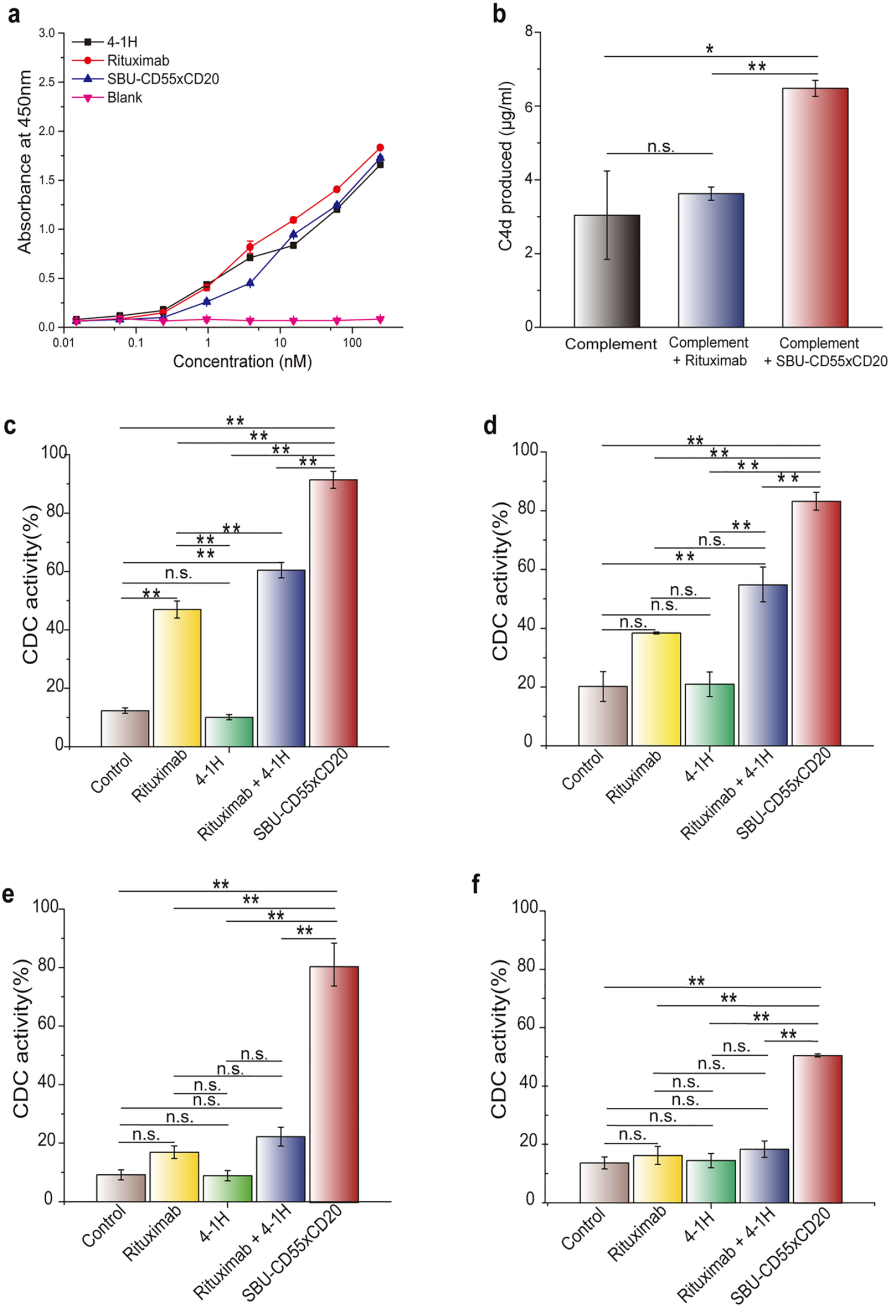
C1q binding, C4d production, and serum complement–mediated tumor cell killing of SBU-CD55 × CD20. (**a**) Binding of SBU-CD55 × CD20, rituximab, and 4-1H to C1q detected by ELISA. (**b**) C4d production of SBU-CD55 × CD20. C4d produced in the presence of serum complements was measured using a C4d ELISA kit, and its signals were compared with those of rituximab. (**c**–**f**) CDC of SBU-CD55 × CD20, rituximab, 4-1H, and the combination of two IgG antibodies (rituximab and 4-1H) in lymphoma cell lines: Ramos (**c**), WSU-NHL (**d**), Ramos-RR (**e**), and BJAB (**f**). Standard errors calculated from triplicate samples are represented by error bars. Standard errors calculated from triplicate samples are represented by error bars. A *P*-value < 0.01 is represented as **, and a *P*-value > 0.05 is indicated by “n.s.” for not significant.

SBU-CD55 × CD20, which is smaller than a natural IgG antibody (~ 125 kDa *vs.* ~ 150 kDa), has flexible linkers and Knob-into-Hole mutations in the antigen-binding and Fc regions, respectively. To investigate the effect of these structural differences from native IgG molecules on the function of the Fc domain, we analyzed the binding affinity of SBU-CD55 × CD20 to the Fc binding ligands human FcγRs, human FcRn, and human C1q. ELISA results showed that SBU-CD55 × CD20 exhibited binding to all human FcγRs and pH-dependent binding to human FcRn, almost identical to that of rituximab and 4-1H (Supplementary Fig. S7). With respect to C1q binding, SBU-CD55 × CD20 showed slightly lower ELISA binding signals than rituximab and 4-1H (Fig. 4a). On the other hand, SBU-CD55 × CD20 showed higher production of C4d molecules than rituximab (Fig. 4b). Because heterogeneous glycosylation profiles may cause differences in the structure and function of antibodies, we performed *N*-linked glycan profile analysis using mass spectrometry. The results revealed that the glycan profile of the SBU-CD55xCD20 antibody was highly similar to that of rituximab in terms of major glycan forms such as GOF, G1F, and G2F (Supplementary Fig. S8a). In a thermostability analysis using a DSC instrument, SBU-CD55 × CD20 showed a high melting temperature (T_m_1 = 68.77 °C), which was comparable to that of rituximab IgG (T_m_1 = 72.61 °C) (Supplementary Fig. S8b). Taken together, the results of the physicochemical analyses suggest that the SBU-CD55 × CD20 antibody has excellent developability as a therapeutic bispecific antibody.

### SBU-CD55 × CD20 elicits higher CDC than an anti-CD20 IgG antibody, anti-CD55 IgG antibody, or the combination of the two monospecific IgG antibodies

Although rituximab has been used to treat non-Hodgkin’s lymphoma and chronic lymphocytic leukemia since its FDA approval in 1997, relapsed or refractory cancers resistant to rituximab have been reported^37,38^. When resistance develops and relapse occurs, the available drugs are very limited; therefore, new treatments are needed to overcome the limitations of existing therapies.

CD55 inhibits CDC by accelerating C3 convertase degradation^39^. To analyze the CDC of SBU-CD55 × CD20, which has an asymmetric structure capable of binding to CD55 and CD20 simultaneously, we used two rituximab-sensitive cell lines (Ramos and WSU-NHL) and two rituximab-resistant cell lines (in-house developed Ramos-RR and BJAB). Rituximab-sensitive cell lines (Ramos and WSU-NHL) showed CDC of 47% and 38%, respectively, and 4-1H (anti-CD55 antibody) showed CDC-induced tumor cell clearance of 10% and 21%, respectively. The combination of rituximab and 4-1H improved CDC to 60% and 54.72% in Ramos and WSU-NHL cells, respectively. In sharp contrast, the SBU-CD55 × CD20 antibody exhibited dramatically improved tumor cell clearance of 91.42% and 83.21% in the Ramos and WSU-NHL cells, respectively (Fig. 4c,d). In the rituximab-resistant cell lines (Ramos-RR and BJAB cells), the CDC of each monospecific antibody (rituximab or 4-1H antibody) was very low (8.89–16.91%), and the combination of the two monospecific antibodies (rituximab and 4-1H) also had low tumor cell-killing activity of 22.24% and 18.34% in Ramos-RR and BJAB cells, respectively. In sharp contrast, the SBU-CD55 × CD20 antibody exhibited a remarkably increased CDC of 83% and 50.49% in Ramos-RR and BJAB, respectively (Fig. 4e,f), indicating its superior tumor cell killing efficacy in resistant tumor cell lines.

### Comparison of CDC between the SBU and bispecific antibodies with different forms

**Figure 5:**
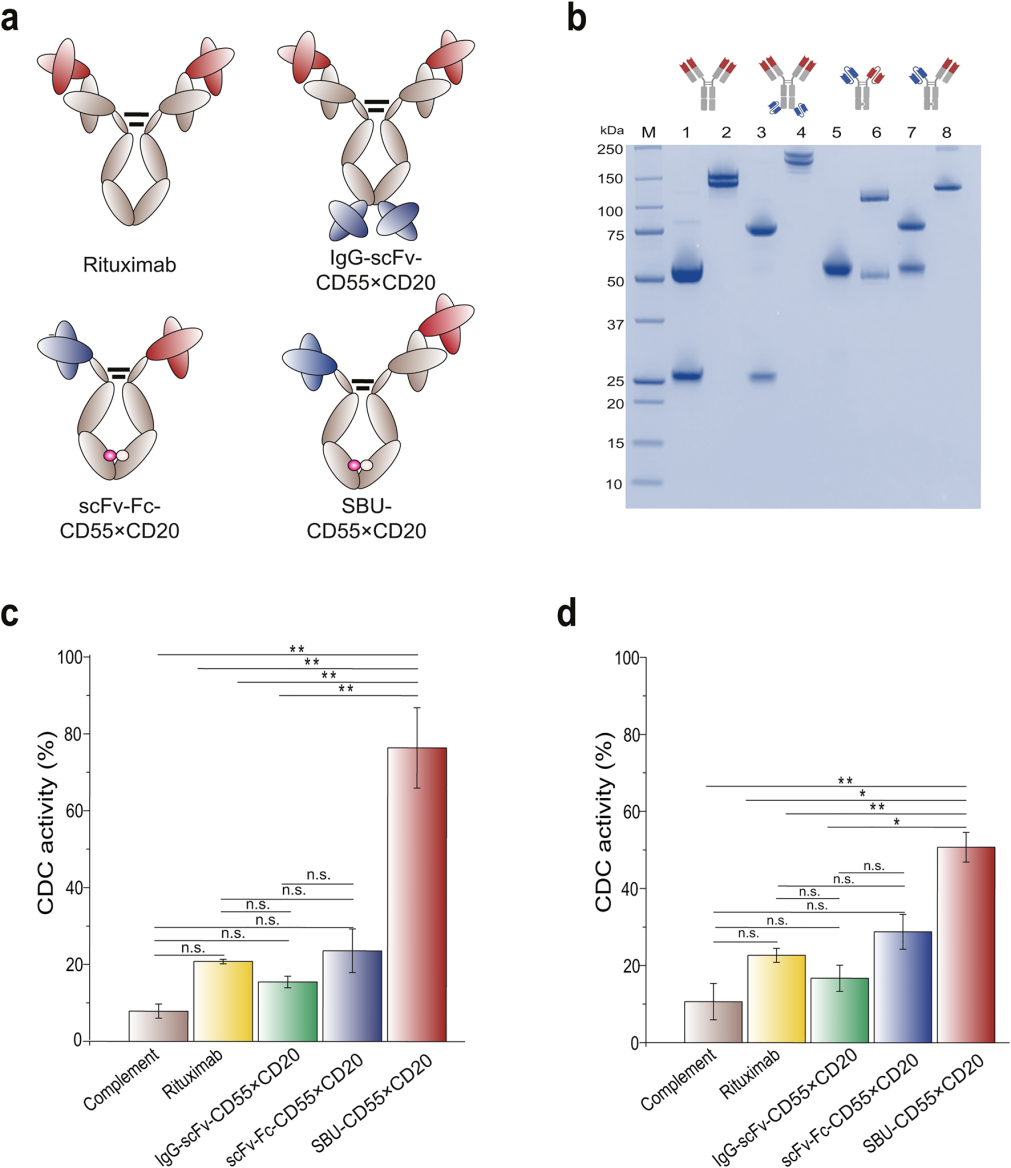
Production of various antibody forms and their CDC in a rituximab-resistant cell line. (**a**) Schematic diagrams for the expression of the SBU-CD55 × CD20 and control antibodies (rituximab, IgG-scFv-CD55 × CD20, and scFv-Fc-CD55 × CD20). (**b**) SDS-PAGE analysis showing the purified SBU-CD55 × CD20 and control antibodies (rituximab, IgG-scFv-CD55 × CD20, and scFv-Fc-CD55 × CD20) in reduced and nonreduced conditions. Lane 1: reduced rituximab; Lane 2: nonreduced rituximab; Lane 3: reduced IgG-scFv-CD55 × CD20; Lane 4: nonreduced IgG-scFv-CD55 × CD20; Lane 5: reduced scFv-Fc-CD55 × CD20; Lane 6: nonreduced scFv-Fc-CD55 × CD20; Lane 7: reduced SBU-CD55 × CD20; Lane 8: nonreduced SBU-CD55 × CD20. (**c**,**d**) CDC of SBU-CD55 × CD20 in rituximab-resistant cell lines (Ramos-RR and BJAB). Ramos-RR (**c**) and BJAB (**d**) cells were used to measure the CDC of SBU-CD55 × CD20 and three control antibodies (rituximab, IgG-scFv-CD55 × CD20, and scFv-Fc-CD55 × CD20). Error bars represent standard errors calculated from triplicate samples. *P*-values < 0.05, < 0.01, and > 0.05 are marked with *, **, and “n.s.” for not significant, respectively.

Several different forms of bispecific antibodies have been developed to control two different antigens simultaneously^6^. We prepared different forms of bispecific antibodies and compared their efficacies with that of SBU-CD55 × CD20. In contrast to SBU-CD55 × CD20 (scFv-Fc_Knob_–scFab-Fc_Hole_), where the CD55 and CD20 antigen-binding sites of the antibody were scFv and scFab, respectively, we prepared two different forms of bispecific antibodies: (1) scFv-Fc-CD55 × CD20 (scFv-Fc_Knob_–scFv-Fc_Hole_), comprising two scFvs against CD55 and CD20, respectively, and an Fc region with a Knob mutation (T366W) and hole mutations (T366S, L368A, and Y407), and (2) IgG-scFv-CD55 × CD20, which had an anti-CD55 scFv fused to the C-terminus of the heavy chain of rituximab IgG (Fig. 5a). When the three forms of bispecific antibodies (SBU-CD55 × CD20, scFv-Fc-CD55 × CD20, and IgG-scFv-CD55 × CD20) were expressed in Expi293F cells, purified with KappaSelect resin or Protein A resin, and analyzed by SDS-PAGE, bands corresponding to the expected molecular weights of the three formats of bispecific antibodies (rituximab IgG,150 kDa; SBU-CD55 × CD20,125 kDa; scFv-Fc-CD55 × CD20,100 kDa; IgG-scFv-CD55 × CD20,200 kDa) were detected (Fig. 5b). In contrast to the IgG-scFv-CD55 × CD20, which showed two bands (~ 200 kDa), and the scFv-Fc-CD55 × CD20, which showed an impurity band (~ 50 kDa) in the SDS-PAGE analysis, possibly due to glycan heterogeneity and non-heterodimerized products (scFv-Fc), respectively, we confirmed that SBU-CD55 × CD20 was clearly purified (Fig. 5b).

The CDC of rituximab IgG and three forms of anti-CD55 × CD20 bispecific antibodies were then analyzed in the two rituximab-resistant cell lines (Fig. 5c,d). Compared with the CDC of rituximab, IgG-scFv-CD55 × CD20 showed 0.72- and 0.75-fold CDC in BJAB and Ramos-RR cells, respectively, indicating decreased tumor cell killing activity; scFv-bsAb showed 1.27- and 1.15-fold CDC in BJAB and Ramos-RR cells, respectively, indicating similar or slightly enhanced CDC. In contrast, SBU-CD55 × CD20 showed 2.7- and 3.8-fold CDC in BJAB and Ramos-RR cells, respectively, which was a significant improvement resulting in 50% and 76% tumor cell lysis, respectively. Taken together, these results demonstrate that CDC is highly variable depending on the form of the bispecific antibody, and the asymmetric bispecific antibody (SBU) demonstrated efficacy that was highly superior to that of the other forms in killing tumor cells, as well as having advantages from a bioprocessing standpoint.

## Discussion

To improve CDC, researchers have worked to enhance the Fc–C1q interaction or to facilitate hexamerization of IgG molecules by engineering the Fc region^15,16,18,40^. In addition, the structural characteristics of the antibody Fab–antigen complex have been shown to affect C1q binding affinity and CDC^21^. Therefore, we hypothesized that an IgG-like antibody in a heterodimer format with an asymmetric structure might induce CDC better than a conventional IgG antibody, and we confirmed that our asymmetric bispecific antibody (SBU) had improved CDC and antitumor effects. In addition, we found that a synergistically enhanced tumor cell-killing effect could be induced by generating an asymmetric bispecific antibody targeting CD20 and CD55, a complement regulatory protein.

The SBU antibody proposed in this study differs from the existing symmetric IgG: (i) antigen-binding fragments of two different sizes (scFv and scFab) and (ii) an asymmetric heterodimer with Knob-into-Hole (KiH) mutations (T366W: T366S, L368A, and Y407) in the Fc region. Since KiH mutations introduced into the Fc region have a negligible effect on CDC (Supplementary Fig. S4b), we believe that the format or bulkiness of the Fabs affects CDC. As a primary step in the initiation of CDC, IgG hexamerization facilitates the interaction between the Fc region and the hexameric heads of the C1q molecule^18^, and the degree of IgG hexamerization varies depending on the angle and flexibility between the antibody Fab regions^21^. Therefore, we speculate that the asymmetric structure of the SBU antibody reduces collisions between the antigen-binding arms during hexamerization compared to a native IgG antibody with a symmetric structure (Supplementary Fig. S9). This hypothesis will be elucidated in future work on the biochemical and physicochemical properties of the IgG-C1q complex using native mass spectrometry analysis or structural studies.

Much attention has already been paid to bispecific antibodies in various forms because of their advantages in overcoming the limitations of conventional monoclonal antibodies and enhancing tumor cell-killing activity. Numerous bispecific antibody forms have been developed, including antibody fragment–based bispecific antibodies such as T cell engagers (BiTE from Amgen and DART from Macrogenics) and IgG-like bispecific antibody forms that enable Fc-mediated effector functions and prolong circulating half-life^6^. However, from a bioprocessing standpoint, several issues remain to be addressed, such as mispairing between antibody heavy and light chains, low productivity, and complex purification steps. We have used Knob-into-Hole mutations^41,42^ and flexible linkers for efficient inter-heavy chain heterodimerization and correct antibody heavy/light chain pairings, respectively. Because the SBU contains a Cκ light chain domain on only one antigen-binding arm, a highly purified bispecific antibody could be obtained by a single step of affinity purification (Supplementary Fig. S1), indicating that an SBU with an asymmetric structure is an effective format for the simple production of bispecific antibodies.

Despite the use of rituximab in combination with CHOP (cyclophosphamide, doxorubicin, vincristine, and prednisone) for the treatment of B-cell lymphoma, many patients develop resistance to rituximab and suffer from refractory or relapsed lymphomas. To identify the causes of rituximab resistance, we developed a rituximab-resistant cell line and found that its expression of CD20 decreased while its expression of CD55 increased significantly. Our finding that CD55 overexpression and CD20 antigen loss are highly correlated with rituximab resistance is in good agreement with previous results^43^. Based on these findings, we developed an asymmetric bispecific antibody targeting both CD20 and CD55 to overcome the limitations of rituximab.

Our bispecific antibody with broken mirror-symmetry (SBU-CD55 × CD20) showed superior CDC and higher tumor cell clearance activity than the combination of rituximab and 4-1H and either agent alone. The mechanism by which asymmetric bispecific antibodies enhance CDC beyond the combination of the two parental monoclonal IgG antibodies is not yet clear at the molecular level. Kumar et al. reported that three anti-CD20 antibodies (rituximab, ofatumumab, and obinutuzumab) have different interarm angles^21^. Thus, considering that rituximab and 4-1H may have different geometries, the combination of two antibodies (rituximab and 4-1H) is thought to have produced a less ordered arrangement of antigen–antibody complexes than the use of a single agent (SBU-CD55 × CD20) with an asymmetric structure. Therefore, the homogeneity of the antigen–antibody complexes is likely to cause differences in antibody hexamerization and CDC (Supplementary Fig. S10a–c).

Santich et al. reported that the antitumor efficacy of a T cell-engaging bispecific antibody varies depending on the spatial configuration of the antibody^44^. Similarly, our results strongly suggest that the shape of a bispecific antibody is critical to its CDC, thus it is necessary to establish an optimal engineering strategy that includes in-depth consideration of therapeutic modalities and therapeutic windows when designing a bispecific antibody.

In this study, we hypothesized that breaking the symmetry of an antibody improved CDC and that its cancer cell-killing effect was synergistically enhanced by formulating a bispecific antibody targeting CD55, an immune checkpoint inhibitor of the complement cascade in the human immune system. Our asymmetric bispecific antibody that enhances CDC could be used as an innovative therapeutic agent for the treatment of relapsed or refractory cancers that are resistant to existing anticancer drugs, including rituximab.

## Materials and methods

### Reagents

All oligonucleotide primers are summarized in Supplementary Table S1 and were synthesized by Cosmogentech. Vent polymerase, restriction enzymes, and T4 DNA ligase (Cat. No. M0202M) were purchased from New England Biolabs. The B-cell lymphoma (WSU-NHL) and Burkitt lymphoma (BJAB) cell lines (Cat. No. ACC 58, ACC 757) were obtained from DSMZ. The Ramos and A549 cell lines (Cat. No. KCLB 21596, KCLB 10,185) were purchased from the Korean Cell Line Bank. Goat anti-human IgG(H + L)-HRP (Cat. No. 109-035-008) and an FITC Annexin V Apoptosis Detection Kit with 7-AAD (Cat. No. 640922) were purchased from Jackson ImmunoResearch and BioLegend, respectively. Protein A (Cat. No. 1010100) and KappaSelect (Cat. No. 17545801) resins were purchased from Amicogen and Cytiva, respectively. Freestyle 293 expression medium (Cat. No. 12338026), Expi293F cells (Cat. No. A14528), 1-Step Ultra TMB solution (Cat. No. 34028), goat anti-human IgG antibody-Alexa Fluor 488 (Cat. No. A-11013), and polypropylene columns (Cat. No. 29922) were purchased from Thermo Fisher Scientific. Lyophilized human serum complement (Cat. No. S1764) and all other chemicals were purchased from Sigma-Aldrich, unless otherwise stated.

### Plasmid construction

The plasmids used in this study are summarized in Supplementary Table S2. To generate FcKnob and FcHole fragments containing a single mutation (T366W) and triple mutations (T366S, L368A, and Y407V), four primers (SM#5–#8) for FcKnob and six primers (SM#13–#18) for FcHole were PCR amplified using pPelB-FLAG-Fc^45^ as a template. The genes encoding single-chain antigen-binding fragments, CD55-scFv, HER2-scFab, CD20-scFab, and EGFR-scFab, were PCR-amplified using sets of primers SM#1–#4, SM#9–#12, SM#21–#24, and SM25#–SM#30, respectively. The amplified PCR fragments for FcKnob and CD55-scFv were PCR-assembled using two primers (SM#1 and SM#8) to generate CD55-scFv-FcKnob. Similarly, HER2-scFab-FcHole, CD20-scFab-FcHole, and EGFR-scFab-FcHole were constructed using primer sets SM#9/SM#18, SM#21/SM#8, and SM25#/SM#8, respectively. In addition, CD20-scFv-FcKnob and CD20-HC-CD55-scFv were PCR assembled using primers SM#31–#35/SM#8 and SM#36–#39, respectively. The resulting amplified PCR products were ligated into pMAZ-IgL^45,46^ using *Xba*I/*Bss*HII restriction enzyme sites for transformation into *E. coli* Jude1 (*F' [Tn10*(*Tet*^r^) *proAB*^+^* lacI*^q^ *Δ*(*lacZ)M15] mcrA Δ(mrr-hsdRMS-mcrBC*) *80dlacZΔM15 ΔlacX74* *deoR* *recA1* *araD139* *Δ(ara leu)7697* *galU* *galK* *rpsL* *endA1* *nupG*)^47^.

### Expression and purification of antibodies

Plasmids encoding antibody heavy and light chains were transfected into Expi293F cells adapted for growth in suspension culture in Freestyle293 expression medium. Purified plasmids (150 μg) for each bispecific antibody heavy and light chains were added to an aliquot of culture medium equivalent to 10% of the final culture volume and incubated at room temperature for 5 min. After the addition of 1.2 ml of filter-sterilized polyethylenimine (PEI) solution (1 mg/ml, pH 7.2), the PEI-DNA mixture was incubated at room temperature for 20 min, and the mixture was added to the cell culture suspension for transfection at 37 °C with 8% CO_2_ and shaking at 135 rpm for 7 days. The supernatants were recovered by centrifugation at 6000 × g for 15 min. After filtration of the supernatants through a 0.22 μm bottle-top filter, the filtrate was incubated with 1 ml of KappaSelect pre-equilibrated in 1 × phosphate buffered saline (PBS) buffer (pH 7.4) for 16 h at 4 °C. After washing in 10 ml of 1 × PBS (pH 7.4), the bispecific antibodies were eluted in 5 ml of 0.1 M glycine (pH 2.7), neutralized the eluates by adding 500 μl of 1 M Tris (pH 8.0), and then concentrated on Amicon Ultra-4 centrifugal filter units (EMD Millipore).

### ELISA

We coated a 96-well polystyrene ELISA plate (Costar) with 50 μl of 4 μg/ml antigen (CD55 or HER2/CD20/EGFR) resuspended in 0.05 M Na2CO3 (pH 9.6) in for coating and then incubated the plate overnight at 4 °C. After blocking each well of the plate with 100 μl of 1 × PBS and 4% skim milk, the plate was incubated at room temperature for 1 h, washed four times with 1 × PBS containing 0.05% Tween20 (0.05% PBST), and then 50 μl of serially diluted, purified bispecific antibodies was added. The plate was then incubated for 1 h at room temperature, washed four times with 0.05% PBST, and treated with 50 μl of 1:5000 diluted goat anti-human IgG (H + L)-HRP conjugate. After another hour of incubation at room temperature and four washes in 0.05% PBST, ELISA signals were developed by adding 50 μl of 1-Step™ Ultra-TMB solution and incubating the plate at room temperature. Next, 50 μl of 2 M H2SO4 was added to each well to quench the signals, and the absorbance was measured at 450 nm using a plate reader (BioTek).

### Size exclusion chromatography (SEC) analysis

The bispecific antibodies were separated using a Waters Alliance 2695 system (Milford) with a BioSuite High Resolution (7.5 mm × 300 mm, 10 μm particle size) size-exclusion column. Separation was performed using an isocratic flow and 1 × PBS buffer, pH 7.4. The injection concentration and volume were 1 mg/ml and 10 μl, respectively.

### Reverse phage chromatography analysis

Reverse phase (RP)-HPLC was performed on an Agilent 1260 Infinity system (Agilent, Santa Clara) using a Waters Xbridge BEH300 C4 column (4.6 × 150 mm, 3.5 μm particle size). Separation was performed with eluents A (0.1% trifluoroacetic acid (TFA) in water) and B (0.1% TFA in acetonitrile) with an 18 min linear gradient from 20 to 80% of eluent B at a flow rate of 1.44 ml/min. The injection concentration and volume were 1 mg/ml and 10 μl, respectively.

### Thermostability analysis using differential calorimetry

The Tm values of the bispecific antibodies were measured using a differential scanning calorimetry system (DSC, TA Instruments). We loaded 800 μl of the antibodies at a concentration of 1 mg/ml and raised the temperature from 10 to 100 °C at a heating rate of 1 °C/min. The equilibration time was set to 600 s. Buffer subtraction and baseline integration were performed after Tm analysis using a two-state scaled model.

### N-linked glycan profiling

Glycan profiling was performed as described previously^48, 49^. Briefly, the glycans released from 25 μl of the antibody were labeled and enriched sing a Rapi-Fluor labeling kit, and the resulting products were analyzed on a Waters ACQUITY I class system with an ACQUITY UPLC Glycan BEH amide column (2.1 mm × 150 mm, 1.7 μm particle size). Separation was performed using eluent A (50 mM ammonium formate, pH 4.5) and eluent B (acetonitrile) with a 35-min linear gradient from 25 to 46% of eluent B at a flow rate of 0.4 ml/min. The elent was detected by excitation and emission at 264 nm and 425 nm, respectively.

### Analysis of cell-surface antigen binding

Ramos and A549 cells expressing CD20 and CD55, respectively, were maintained in RPMI 1640 supplemented with 10% fetal bovine serum FBS (Thermo Fisher Scientific). To analyze the binding between the prepared antibody and the cell surface antigens, 1 × 10^6^ cells resuspended in 1% bovine serum albumin/PBS with or without 10 μg/ml of SBU-CD20 were incubated at 4 °C for 1 h, pelleted by centrifugation at 1500 × *g* for 5 min, and washed in 1 × PBS. Next, 100 μl of FITC-conjugated goat anti-human IgG antibody was added, and the cells were incubated for 1 h at 4 °C in the dark. After washing in cold 1 × PBS, the stained cells were analyzed using an Attune NxT flow cytometer (Thermo Fisher Scientific). We used rituximab (anti-CD20) and 4-1H (anti-CD55) were used as control antibodies for CD20 and CD55 binding, respectively.

### Construction of rituximab-resistant cell line

Rituximab-resistant cells were generated from Ramos cells maintained in RPMI 1640 supplemented with 10% FBS at 37 °C and 5% CO_2_. Wild-type Ramos cells were exposed to rituximab (0.125–8 μg/ml) for 24 h and incubated in fresh medium for 72 h. The recovered cells were then treated with rituximab twice a week, with the rituximab concentration doubling in each experiment.

### CDC assays

CDC assays were performed as previously described^35^, with some modifications. Briefly, the target cell lines, Ramos, Ramos-RR, BJAB, and WSU-NHL, were maintained in RPMI 1640 supplemented with 10% FBS. Subsequently, 1 × 10^5^ target cells resuspended in 20 μl of human serum complement and 80 μl of serum-free RPMI 1640 were incubated with or without 20 μg/ml rituximab, rituximab-KiH, SBU-CD20, 4-1H, and/or SBU-CD55 × CD20 at 37 °C for 2 h. Dead and viable cells were analyzed by fluorescence-activated cell sorting (FACS) using a FITC Annexin V apoptosis detection kit with 7-AAD (BioLegend). Complement activation was measured using a MicroVue C4d ELISA kit (Quidel) with the supernatants obtained from the CDC analysis. Each sample was diluted 70-fold with a Complement Specimen Diluent (Quidel), and ELISA analysis was performed according to the manufacturer’s protocol.

### Statistical analysis

All the data are presented as mean ± standard error. Statistical significance was determined using one-way ANOVA for comparisons among multiple groups, followed by post hoc Tukey’s test. A *P*-value of less than 0.05 was considered to indicate statistical significance.

### Supplementary Information

Supplementary Information.

## Data Availability

The datasets generated during and/or analyzed during the current study are available from the corresponding author on reasonable request.

## References

[1] Zhang X (2015). 3D structural fluctuation of IgG1 antibody revealed by individual particle electron tomography. Sci. Rep..

[2] Saphire EO (2001). Crystal structure of a neutralizing human IGG against HIV-1: A template for vaccine design. Science..

[3] Shah A (2021). The current landscape of antibody-based THERAPIES IN SOLID MALIGNANCIES. Theranostics..

[4] Kaplon H, Chenoweth A, Crescioli S, Reichert JM (2022). Antibodies to watch in 2022. MAbs..

[5] *Antibody Society*, [cited 2022 November 1]. https://www.antibodysociety.org/resources/approved-antibodies/.

[6] Labrijn AF, Janmaat ML, Reichert JM, Parren P (2019). Bispecific antibodies: A mechanistic review of the pipeline. Nat. Rev. Drug Discov..

[7] Brinkmann U, Kontermann RE (2021). Bispecific antibodies. Science..

[8] Ma J (2021). Bispecific antibodies: From research to clinical application. Front. Immunol..

[9] Esfandiari A, Cassidy S, Webster RM (2022). Bispecific antibodies in oncology. Nat. Rev. Drug Discov..

[10] Park HI, Yoon HW, Jung ST (2016). The highly evolvable antibody Fc domain. Trends Biotechnol..

[11] Kang TH, Jung ST (2019). Boosting therapeutic potency of antibodies by taming Fc domain functions. Exp. Mol. Med..

[12] Kang, T. H. & Jung, S. T. Reprogramming the constant region of immunoglobulin G subclasses for enhanced therapeutic potency against cancer. *Biomolecules*. **10** (2020). 10.3390/biom10030382 PMC7175108 32121592

[13] Beers SA, Chan CHT, French RR, Cragg MS, Glennie MJ (2010). CD20 as a target for therapeutic type I and II monoclonal antibodies. Semin. Hematol..

[14] Cragg MS, Glennie MJ (2004). Antibody specificity controls in vivo effector mechanisms of anti-CD20 reagents. Blood..

[15] Moore GL, Chen H, Karki S, Lazar GA (2010). Engineered Fc variant antibodies with enhanced ability to recruit complement and mediate effector functions. MAbs..

[16] Idusogie EE (2001). Engineered antibodies with increased activity to recruit complement. J. Immunol..

[17] Peschke B, Keller CW, Weber P, Quast I, Lunemann JD (2017). Fc-galactosylation of human immunoglobulin gamma isotypes improves C1q binding and enhances complement-dependent cytotoxicity. Front. Immunol..

[18] Diebolder CA (2014). Complement is activated by IgG hexamers assembled at the cell surface. Science..

[19] Tammen A (2017). Monoclonal antibodies against epidermal growth factor receptor acquire an ability to kill tumor cells through complement activation by mutations that selectively facilitate the hexamerization of IgG on opsonized cells. J. Immunol..

[20] de Jong RN (2016). A novel platform for the potentiation of therapeutic antibodies based on antigen-dependent formation of IgG hexamers at the cell surface. PLoS Biol..

[21] Kumar A, Planchais C, Fronzes R, Mouquet H, Reyes N (2020). Binding mechanisms of therapeutic antibodies to human CD20. Science..

[22] Pawluczkowycz AW (2009). Binding of submaximal C1q promotes complement-dependent cytotoxicity (CDC) of B cells opsonized with anti-CD20 mAbs ofatumumab (OFA) or rituximab (RTX): considerably higher levels of CDC are induced by OFA than by RTX. J. Immunol..

[23] Dho, S. H., Lim, J. C. & Kim, L. K. Beyond the role of CD55 as a complement component. *Immune Netw*. **18** (2018). 10.4110/in.2018.18.e11 PMC5833118 29503741

[24] Dunkelberger JR, Song WC (2010). Complement and its role in innate and adaptive immune responses. Cell Res..

[25] Ikeda J (2008). Prognostic significance of CD55 expression in breast cancer. Clin. Cancer Res..

[26] Hara T (1992). Levels of complement regulatory proteins, CD35 (CR1), CD46 (MCP) and CD55 (DAF) in human haematological malignancies. Br. J. Haematol..

[27] Nakagawa M (2001). Polymorphic expression of decay-accelerating factor in human colorectal cancer. J. Gastroenterol. Hepatol..

[28] Inoue T, Yamakawa M, Takahashi T (2002). Expression of complement regulating factors in gastric cancer cells. Mol. Pathol..

[29] Macor P (2015). Bispecific antibodies targeting tumor-associated antigens and neutralizing complement regulators increase the efficacy of antibody-based immunotherapy in mice. Leukemia..

[30] Wang Y (2017). CD55 and CD59 expression protects HER2-overexpressing breast cancer cells from trastuzumab-induced complement-dependent cytotoxicity. Oncol. Lett..

[31] Merchant AM (1998). An efficient route to human bispecific IgG. Nat. Biotechnol..

[32] Fischer, N. *et al*. Exploiting light chains for the scalable generation and platform purification of native human bispecific IgG. *Nat. Commun*. **6** (2015). 10.1038/ncomms7113 PMC4339886 25672245

[33] Nilson BHK, Solomon A, Bjorck L, Akerstrom B (1992). Protein-L from Peptostreptococcus-magnus binds to the kappa-light chain variable domain. J. Biol. Chem..

[34] Klein C (2013). Epitope interactions of monoclonal antibodies targeting CD20 and their relationship to functional properties. Mabs..

[35] Golay J (2000). Biologic response of B lymphoma cells to anti-CD20 monoclonal antibody rituximab in vitro: CD55 and CD59 regulate complement-mediated cell lysis. Blood..

[36] Dho, S. H. *et al*. Development of a radionuclide-labeled monoclonal anti-CD55 antibody with theranostic potential in pleural metastatic lung cancer. *Sci. Rep*. **8** (2018). 10.1038/s41598-018-27355-8 PMC5997699 29895866

[37] Rezvani AR, Maloney DG (2011). Rituximab resistance. Best Pract. Res. Clin. Haematol..

[38] Smith MR (2003). Rituximab (monoclonal anti-CD20 antibody): mechanisms of action and resistance. Oncogene..

[39] Rogers LM, Veeramani S, Weiner GJ (2014). Complement in monoclonal antibody therapy of cancer. Immunol. Res..

[40] Natsume A (2008). Engineered antibodies of IgG1/IgG3 mixed isotype with enhanced cytotoxic activities. Cancer Res..

[41] Atwell S, Ridgway JB, Wells JA, Carter P (1997). Stable heterodimers from remodeling the domain interface of a homodimer using a phage display library. J. Mol. Biol..

[42] Ridgway JB, Presta LG, Carter P (1996). 'Knobs-into-holes' engineering of antibody CH3 domains for heavy chain heterodimerization. Protein Eng..

[43] Small GW, McLeod HL, Richards KL (2013). Analysis of innate and acquired resistance to anti-CD20 antibodies in malignant and nonmalignant B cells. PeerJ..

[44] Santich, B. H. *et al*. Interdomain spacing and spatial configuration drive the potency of IgG-[L]-scFv T cell bispecific antibodies. *Sci. Transl. Med*. **12** (2020). 10.1126/scitranslmed.aax1315 PMC7437947 32161106

[45] Jung ST (2010). Aglycosylated IgG variants expressed in bacteria that selectively bind FcgammaRI potentiate tumor cell killing by monocyte-dendritic cells. Proc. Natl. Acad. Sci. USA..

[46] Mazor Y, Barnea I, Keydar I, Benhar I (2007). Antibody internalization studied using a novel IgG binding toxin fusion. J. Immunol. Methods..

[47] Kawarasaki Y (2003). Enhanced crossover SCRATCHY: construction and high-throughput screening of a combinatorial library containing multiple non-homologous crossovers. Nucleic Acids Res..

[48] Lauber MA (2015). Rapid preparation of released N-glycans for HILIC analysis using a labeling reagent that facilitates sensitive fluorescence and ESI-MS detection. Anal. Chem..

[49] Lim MS (2019). Validation of Rapi-Fluor method for glycan profiling and application to commercial antibody drugs. Talanta..

