# Supplementary material for: Effective clearance of rituximab-resistant tumor cells by breaking the mirror-symmetry of immunoglobulin G and simultaneous binding to CD55 and CD20
Source: Sci Rep. 2023 Oct 25;13:18275. doi: 10.1038/s41598-023-45491-8 (PMC10600224; doi:10.1038/s41598-023-45491-8)
Supplement: Supplementary file 1 — Supplementary Information. [file 41598_2023_45491_MOESM1_ESM.docx]

**Supplementary Information**

**Effective clearance of rituximab-resistant tumor cells by breaking the mirror-symmetry of Immunoglobulin G and simultaneous binding to CD55 and CD20**

Sang Min Lee^1,2,†^, Sung-Won Min^3,†^, Hyeong Sun Kwon^3^, Gong-Deuk Bae^3^, Ji Hae Jung^3^, Hye In Park^3^, Seung Hyeon Lee^1^, Chung Su Lim^4^, Byoung Joon Ko^5^, Ji Chul Lee^3,^**^*^**, and Sang Taek Jung^1,6,7,8,^**^*^**

*^1^Department of Biomedical Sciences, Graduate School of Medicine, Korea University, 73 Goryeodae-ro, Seongbuk-gu, Seoul, 02841, Republic of Korea*

*^2^Department of Applied Chemistry, Kookmin University, 77, Jeongneung-ro, Seongbuk-gu, Seoul, 02707, Republic of Korea*

*^3^SG Medical, 3-11, Ogeum-ro 13-gil, Songpa-gu, Seoul, 05548, Republic of Korea*

*^4^New Drug Development Center, Osong Medical Innovation Foundation 123, Cheongju, Chungcheongbuk-do 28160, Republic of Korea*

*^5^School of Biopharmaceutical and Medical Science, Sungshin Women’s University, 55, Dobonng-ro 76ga-gil, Gangbuk, Seoul 01133, Republic of Korea*

*^6^BK21 Graduate Program, Department of Biomedical Sciences, Korea University College of Medicine, Seoul, Republic of Korea*

*^7^Institute of Human Genetics, Korea University College of Medicine, Seoul, 02841, Republic of Korea*

*^8^Biomedical Research Center, Korea University Anam Hospital, Seoul, 02841, Republic of Korea*

†These authors contributed equally to this work.

**Correspondence to: Sang Taek Jung (sjung@korea.ac.kr) or Ji Chul Lee (*[*jclee@sgmedical.kr*](mailto:jclee@sgmedical.kr)*)*

**Supplementary Information Tables**

**Table S1.** Primers used in the current study. Restriction enzyme sites are underlined.

| **Name** | **Primer nucleotide sequence (5`→3`)** |
| --- | --- |
| **SM#1** | CGCAGCGAGCGCGCACTCCGCCCTGACTCAGCCGTCCT |
| **SM#2** | GCCAGATCCACTGCCTCCTCCACCGCTACCGCCACCACCACTCCCGCCTCCGCCTAGGACGGTCAGGGTTGTCCC |
| **SM#3** | GGAGGAGGCAGTGGATCTGGCGGCTCTGTGACGTTGGACGAGTCCGG |
| **SM#4** | CGGTGGGCATGTGTGAGTTTTGTCTGAGCCGCCGGAGGAGACGATGACTTCGGTCC |
| **SM#5** | CCCCCAGCACCTGAAC |
| **SM#6** | CCACAGGCTGACCTGGTTCTTGGTCAGGACATCCAGATGACTCAATCACCCAGTTCAC |
| **SM#7** | CTGACCAAGAACCAGGTCAGCCTGTGGTGCCTGGTCAAAGGCTTCTATCCCGGTCCGCTTAATCTCCACTTTGGTTCC |
| **SM#8** | GGAGGGGCAAACAACAGATGGC |
| **SM#9** | CGCAGCGA GCGCGC ACTCCGATATTCAAATGACCC |
| **SM#10** | CCACCTCCTCCAGAGCCGCCAGACCCGCTACCACCTCCTCCTGAGCCCCCTCCCCCACTTCCTCCGCCCCCGCACTCTCCCCTGTTGAAGCTCT |
| **SM#11** | GCGGCTCTGGAGGAGGTGGGTCCGGTGGTGGAGGGAGTGGTGGTGGAGGCAGCGGATCTGGCTCTAGCGAGGTTCAATTAGTGGAATCTGGCGG |
| **SM#12** | GGAAGACCGATGGGCCCTTGAAGCTTGCAGAGCTGACC |
| **SM#13** | CGGTGGGCATGTGTGAGTTTTGTC |
| **SM#14** | GGCGCAGCTCAGGCTGACCTGGTTCTTGGTCA |
| **SM#15** | CCGGCTGCCCATTGCTCTCCCACTCCACGGCGATGTCGCTGGGATAGAAGCCTTTGACGGCGCAGCTCAGGCTGA |
| **SM#16** | CACGAGGAAGAAGGAGCCGTCGGAGTCCAGCACGGGAGGTGTGGTCTTGTAGTTGTTCTCCGGCTGCCCATTGCTCT |
| **SM#17** | TCACGGAGCATGAGAAGACGTTCCCCTGCTGCCACCTGCTCTTGTCCACGGTGAGCTTGCTCACGAGGAAGAAGGAGCCGTCG |
| **SM#18** | CGCAATTCGGCCCCCGAGGCCTGATGATTTACCCGGGGACAGGGAGAGGCTCTTCTGCGTGTAGTGGTTGTGCAGAGCCTCATGCATCACGGAGCATGAGAAGACGTTCC |
| **SM#19** | ACTACCGGAACCCCCTGATCCGCCGCCTCCGCTTCCGCCACCGCCGCTCCCACCCCCACCAGAGCCGCCAGACCCGC |
| **SM#20** | CGGATCAGGGGGTTCCGGTAGTGGAGGAGGTGGGTCCGGT |
| **SM#21** | CGCAGCGA GCGCGC ACTCC CAGATTGTCCTGTCTCAGTCTCCTGC |
| **SM#22** | CGGCCGCCGTGCGAGATCTTTTGATTTCCAGTTTAGTTCCGCCG |
| **SM#23** | CAAGTCCAACTGCAACAACCGGG |
| **SM#24** | GGAAGACCGATGGGCCCTTGAAGCTAGCGGCGGAAACCGT TGTGCCAGAG |
| **SM#25** | CGCAGCGAGCGCGCACTCCGACATCCTGCTGACCCAGAGC |
| **SM#26** | ATGAAGACAGATGGTGCGGCCACCGTGCGCTTCAGCTCCAGCTTGGTGCC |
| **SM#27** | GGCACCAAGCTGGAGCTGAAGCGCACGGTGGCCGCACCATCTGTCTTCAT |
| **SM#28** | GCTCTGCTTCAGCTGCACCTGGCTAGAGCCAGATCCGCTGC |
| **SM#29** | GCAGCGGATCTGGCTCTAGCCAGGTGCAGCTGAAGCAGAGC |
| **SM#30** | GGGAAGACCGATGGGCCCTTGGTGCTAGCGGCGGACACGG |
| **SM#31** | CGCAGCGAGCGCGCACTCCCAGATCGTCCTGAGTCAGAGCC |
| **SM#32** | AGAGCCCCAGATCCACTGCCTCCTCCACCGCTACCGCCACCACCACTCCCGCCTCCGCCCTTAATCTCCAATTTAGTTCCCCCGC |
| **SM#33** | GCAGTGGATCTGGCGGCTCTCAGGTCCAGCTCCAACAGCCC |
| **SM#34** | GGTGGGCATGTGTGAGTTTTGTCTGAGCCGCCAGCACTCACCGTCACAGTGGTAC |
| **SM#35** | GACAAAACTCACACATGCCCACC |
| **SM#36** | GTAGCAGGCTTGAGGTCTGGACATATATATGG |
| **SM#37** | TTTACCCGGGGACAGGGAGAG |
| **SM#38** | CTCTCCCTGTCCCCGGGTAAAGGCGGTGGGGGATCAGGGGGTGGAGGCAGTGGTGGTGGAGGGAGCGCCCTGACTCAGCCGTCC |
| **SM#39** | TTTTAGGGTCTAGATTATCAGGAGGAGACGATGACTTCGGTC |

**Table S2.** Plasmids used in the present study.

| **Plasmids** | **Characteristics** | **Reference** |
| --- | --- | --- |
| **pMAZ-CD55-scFv-Fc_Knob_** | Amp_r_, *VL (anti-CD55; 4-1H)-GS linker 20-VH (anti CD55; 4-1H)-CH2-CH3 (Knob mutation; 366W)* gene in pMAZ | This study |
| **pMAZ-HER2-scFab-Fc_Hole_** | Amp_r_, *VL (anti-HER2; Trastuzumab)- Cκ -GS linker 40-VH (anti-HER2; Trastuzumab) -CH1-CH2-CH3 (Hole mutations; 366S,368A,407V)* gene in pMAZ | This study |
| **pMAZ-CD20-scFab-Fc_Hole_** | Amp_r_, *VL (anti-CD20; Rituximab)- Cκ -GS linker 40-VH (anti-CD20; Rituximab) -CH1-CH2-CH3 (Hole mutations; 366S,368A,407V)* gene in pMAZ | This study |
| **pMAZ-EGFR-scFab-Fc_Hole_** | Amp_r_, *VL (anti-EGFR; Cetuximab)- Cκ -GS linker 40-VH (cetuximab) -CH1-CH2-CH3 (Hole mutations; 366S,368A,407V)* gene in pMAZ | This study |
| **pMAZ-CD20-scFv-Fc_Knob_** | Amp_r_, *VL (Rituximab)-GS linker 20-VH (Rituximab)-CH2-CH3 (Knob mutation; 366W)* gene in pMAZ | This study |
| **pMAZ-CD20-HC-CD55-scFv** | Amp_r_, *VH-CH1-CH2-CH3 (Rituximab)-GSlinker 15-VL (anti-CD55;4-1H)-GS linker 20-VH (anti-CD55;4-1H)* gene in pMAZ | This study |
| **pMAZ-CD20-LC** | Amp_r_, *Light chain (VL- Cκ ) (anti-CD20; Rituximab)* gene in pMAZ | This study |
| **pMAZ-CD20-HC** | Amp_r_, *Heavy chain (VH-CH1-CH2-CH3) (anti-CD20; Rituximab)* gene in pMAZ | This study |
| **pMAZ-CD20-HC_knob_** | Amp_r_, *Heavy chain (VH-CH1-CH2-CH3) (Knob mutation; 366W) (anti-CD20; Rituximab)* gene in pMAZ | This study |
| **pMAZ-CD20-HC_Hole_** | Amp_r_, *Heavy chain (VH-CH1-CH2-CH3) (Hole mutations; 366S,368A,407V)* *(anti-CD20; Rituximab)* gene in pMAZ | This study |
| **pMAZ-CD20-scFv-Fc_Hole_** | Amp_r_, *VL (Rituximab)-GS linker 20-VH (Rituximab)-CH2-CH3 (Hole mutations; 366S,368A,407V)* gene in pMAZ | This study |
| **ST114** | Cm_r_, pMopac-PelB-Fc-FLAG | [45] |
| **ST470** | Amp_r_, pMAZ-IgL | [45] |

**Supplementary Information Figure Legends**

**Figure S1.** Expression, purification, and RP-analysis of SBU-CD20. (a). SDS-PAGE showing the SBU-CD20 purified using Protein A or KappaSelect affinity chromatography resins. Lane 1: nonreduced SBU-CD20 purified using Protein A resins; Lane 2: nonreduced SBU-CD20 purified using KappaSelect resins. (b and c). RP-HPLC chromatograms for SBU-CD20. SBU-CD20 was analyzed by RP-HPLC after affinity purification using Protein A resins (b) and KappaSelect resins (c).

**Figure S2.** Comparison between the physicochemical properties of SBU-CD20 and those of control antibodies (rituximab and rituximab-KiH). (a). Schematic showing the structure of the control antibodies (rituximab and rituximab-KiH). (b). SDS-PAGE showing purified rituximab and rituximab-KiH. Lane 1: reduced rituximab; Lane 2: nonreduced rituximab; Lane 3: reduced rituximab-KiH: Lane 4: nonreduced rituximab-KiH. (c and d). SEC (c) and RP-HPLC (d) chromatograms for rituximab, rituximab-KiH, and SBU-CD20.

**Figure S3.** Expression and purification of the CD20 epitope (163-187)-fused with streptavidin. (a). Map of plasmid pMAZ-CD20 (163-187)-Streptavidin-His. (b). SDS-PAGE showing the purified CD20 (163-187)-Streptavidin-His. Lane 1: nonreduced CD20 (163-187)-Streptavidin-His; Lane 2: reduced CD20 (163-187)-Streptavidin-His.

**Figure S4.** ELISA showing the binding characteristics of SBU-CD20, rituximab, and rituximab-KiH. (a and b). SBU-CD20, rituximab, and rituximab-KiH binding to CD20 (a) and C1q (b).

**Figure S5.** Schematic representation of the classical complement pathway. The interaction of C1q with the Fc region of IgG triggers a complement cascade, resulting in the generation of membrane attack complexes (MACs). CD55, which accelerates the decay of C3 convertase (C4bC2a) and C5 convertase (C4b2a3b), prevents the reassembly of their components and inhibits MAC formation and CDC.

**Figure S6.** Production and antigen binding characteristics of SBU-CD55×HER2 and SBU-CD55×EGFR. (a). SDS-PAGE showing the purified SBU-CD55×HER2 and SBU-CD55×EGFR. Lane 1: reduced SBU-CD55×HER2; Lane 2: nonreduced SBU-CD55×HER2; Lane 3: reduced SBU-CD55×EGFR; Lane 4: nonreduced SBU-CD55×EGFR. (b and c). ELISA for binding of SBU-CD55×HER2 to CD55 (b) and HER2 (c). (d and e). Binding of SBU-CD55×EGFR to CD55 (d) and EGFR (e), detected by ELISA.

**Figure S7.** Binding characteristics of SBU-CD55×CD20 to human FcγRs and human FcRn. (a–f). ELISA for binding of SBU-CD55×CD20, rituximab, and 4-1H to human FcγRI (a), human FcγRIIa-131R (b), human FcγRIIa-131H (c), human FcγRIIb (d), human FcγRIIIa-158V (e), and human FcγRIIIa-158F (f). (g and h). pH-dependent binding of SBU-CD55×CD20 to human FcRn at pH 7.4 (g) and pH 6.0 (h), detected by ELISA. Error bars represent the standard deviation of two replicates.

**Figure S8.** Physicochemical properties of SBU-CD55×CD20. (a and b). The *N*-linked glycan profile (a) and thermostability (b) of SBU-CD55xCD20 were compared with those of rituximab.

**Figure S9.** (a and b). Proposed models illustrating the interaction of C1q with symmetric IgG (rituximab) (a) and an asymmetric antibody (SBU-CD20) (b).

**Figure S10.** (a–c). Schematic depicting antibodies that bind to cellular membrane–bound antigens when a combination of two monoclonal antibodies (rituximab and 4-1H) (a and b) and SBU-CD55xCD20 (a, c) are administered.

**Figure S11.** CD20 binding affinity saturation curve of Rituximab, Rituximab-KiH, and SBU-CD20 in Ramos cell.

**Figure S12.** SDS-PAGE gel image of original, unprocessed versions. Figure 1c (a), Figure 3c and Figure S6a (b), Figure 5b (c), Figure S1a (d), Figure S2b (e), Figure S3b (f).

**Figure S1.**

**
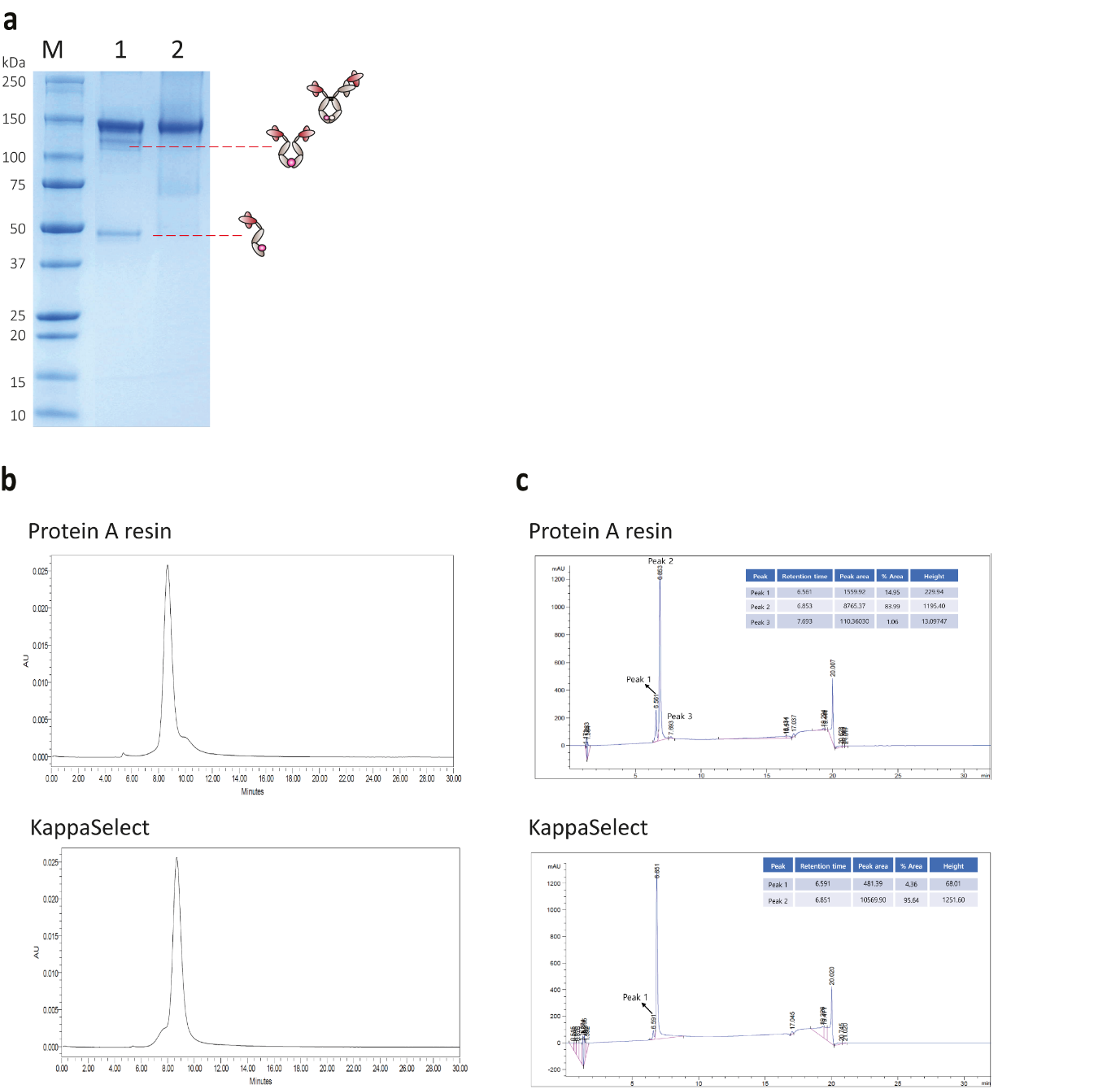
**

**Figure S2.**

**
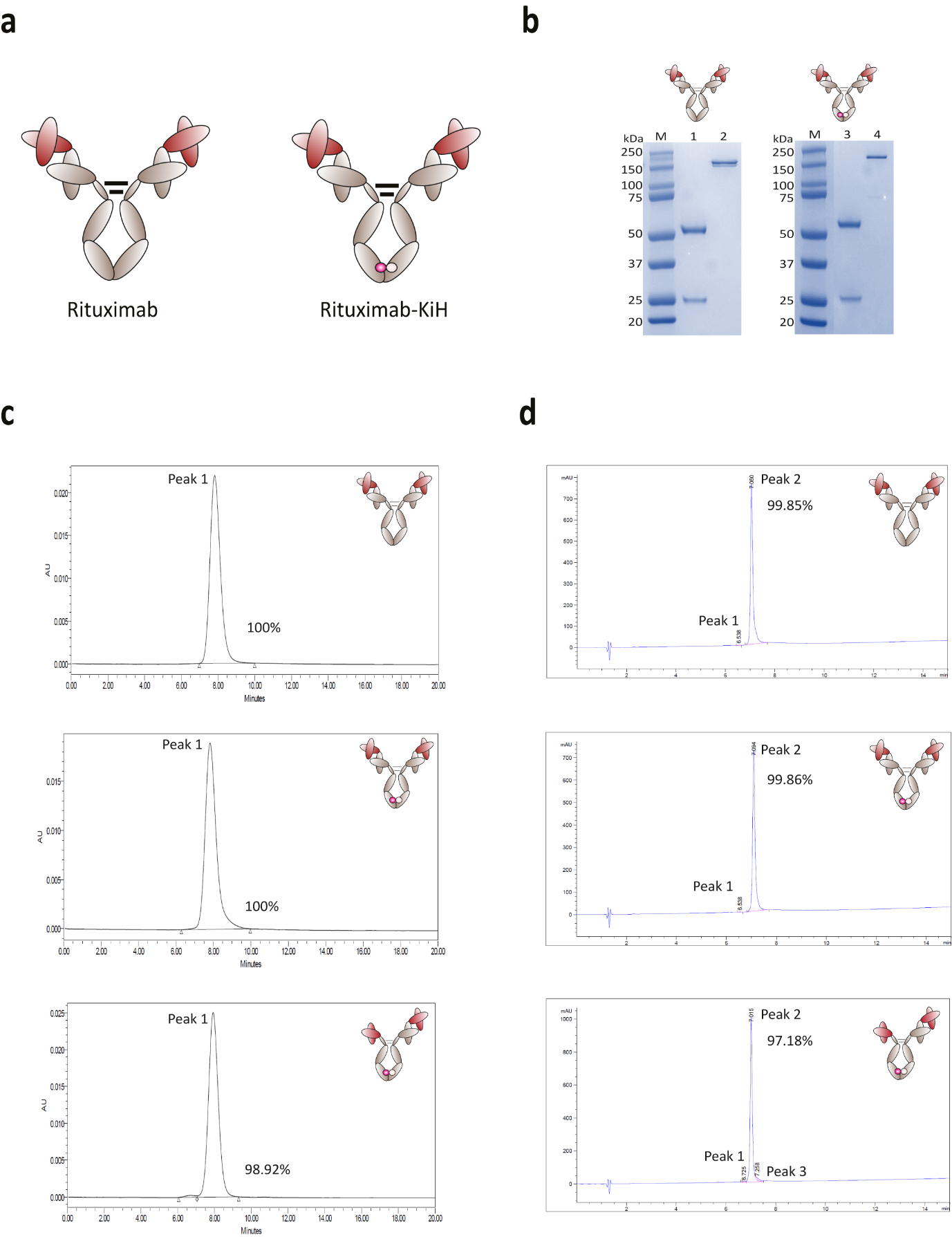
**

**Figure S3.**

**
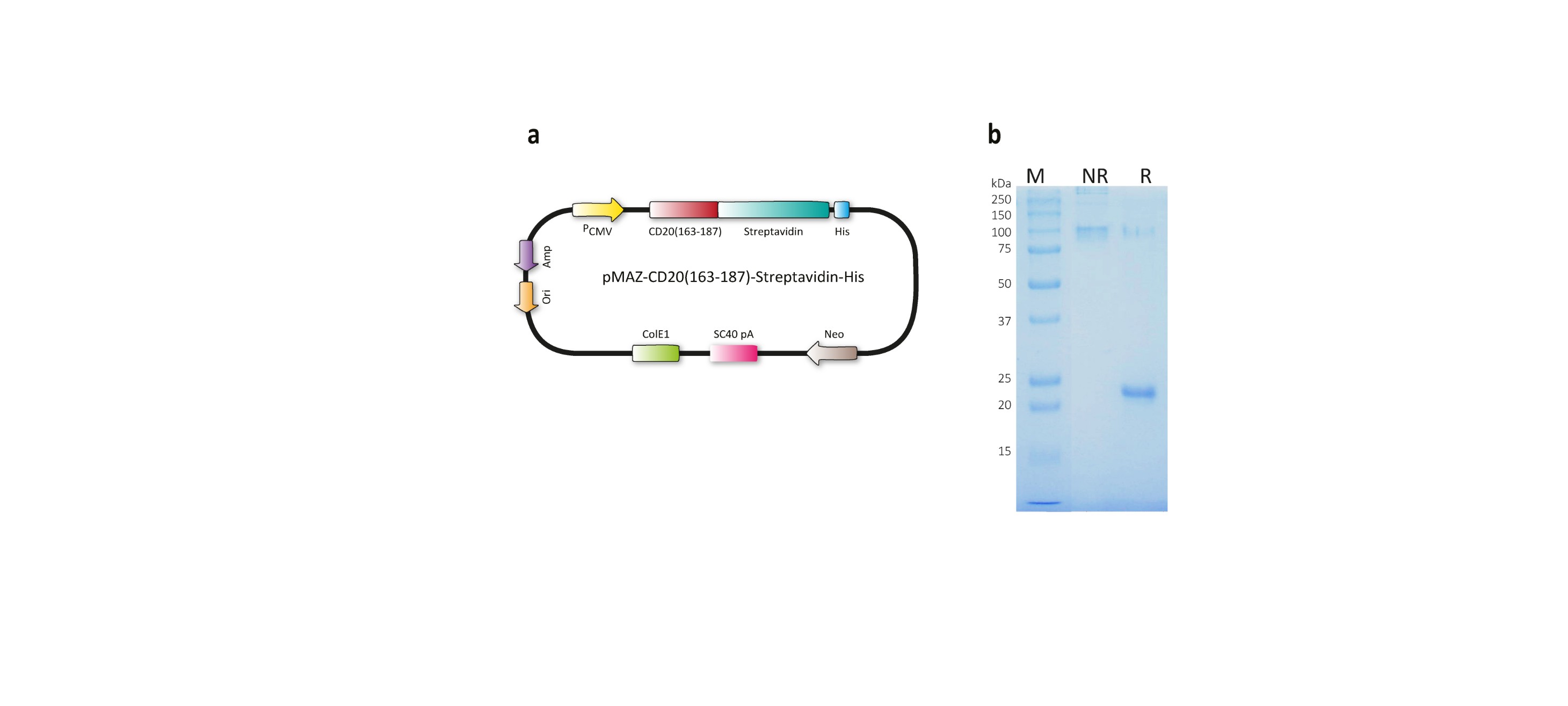
**

**Figure S4.**

**
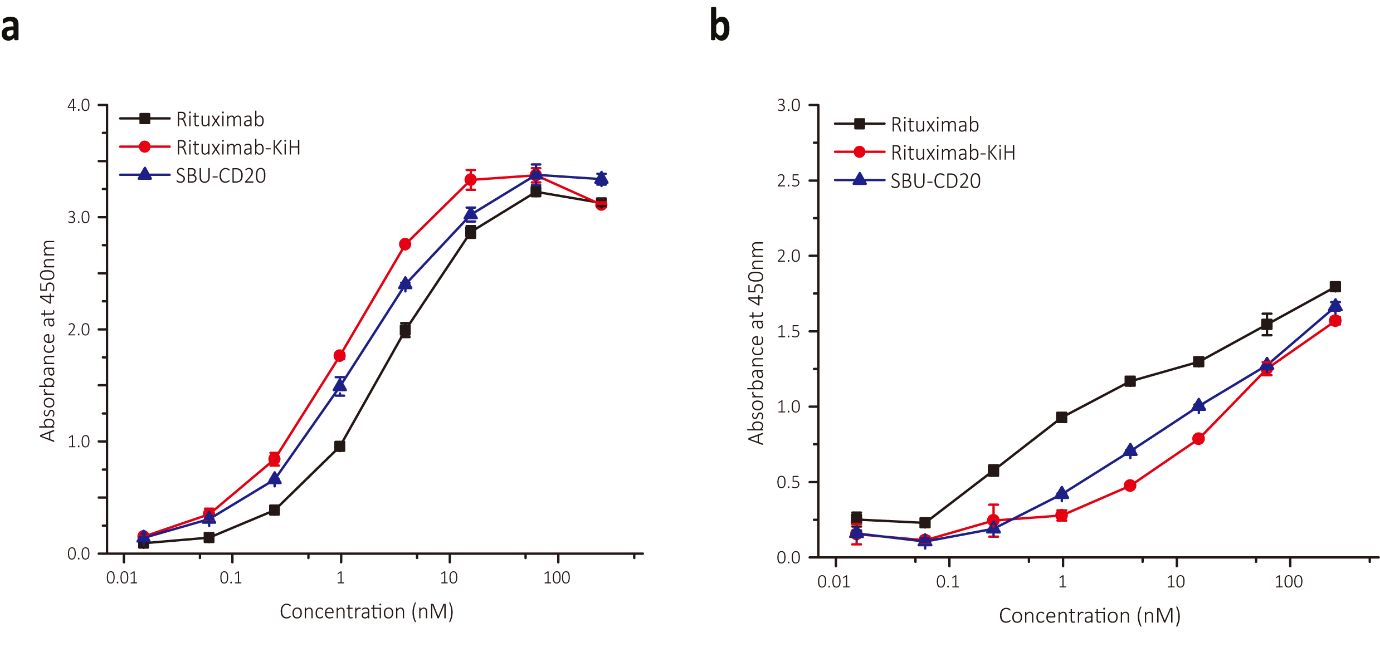
**

**Figure S5.**

**
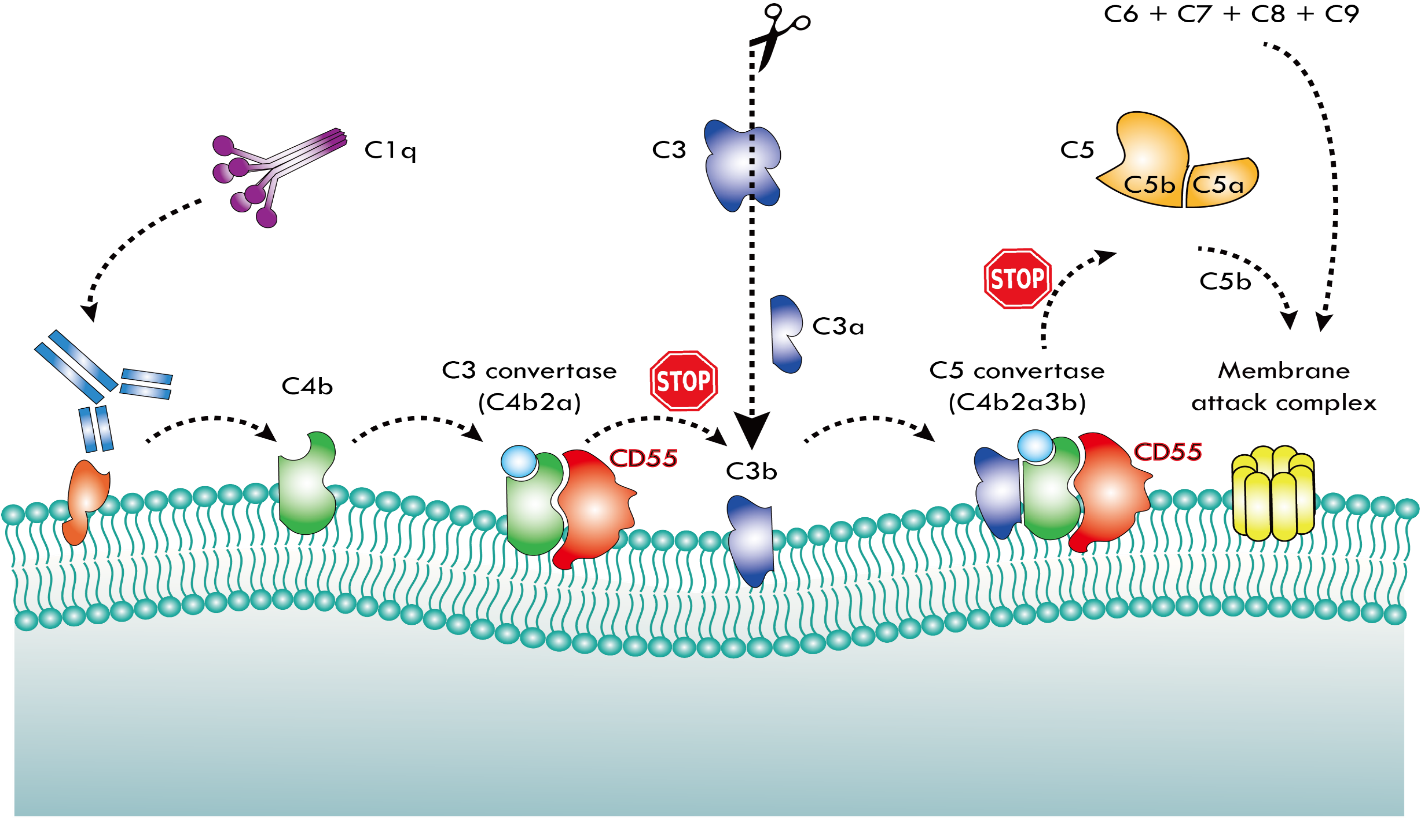
**

**Figure S6.**

**
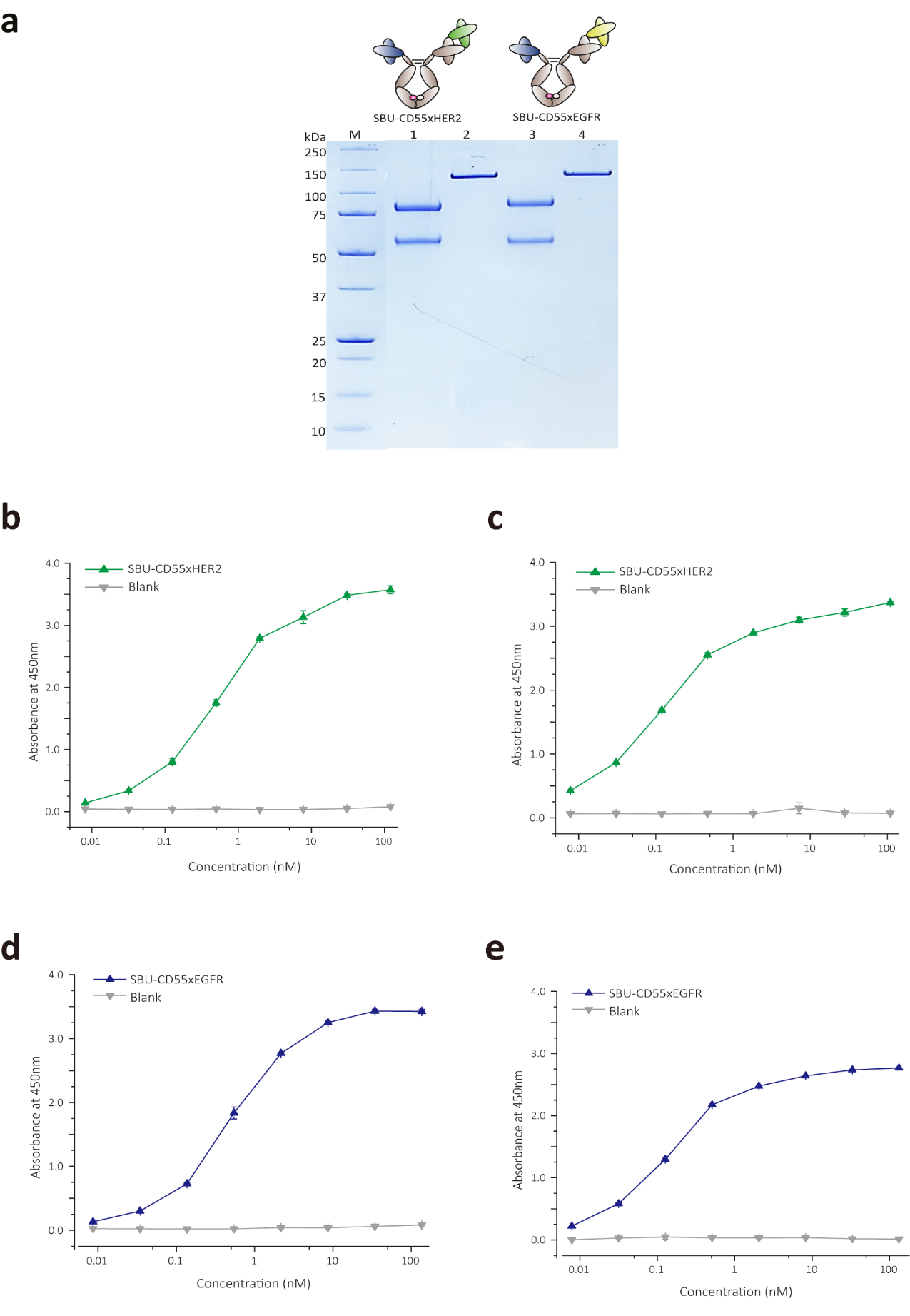
**

**Figure S7.**

**
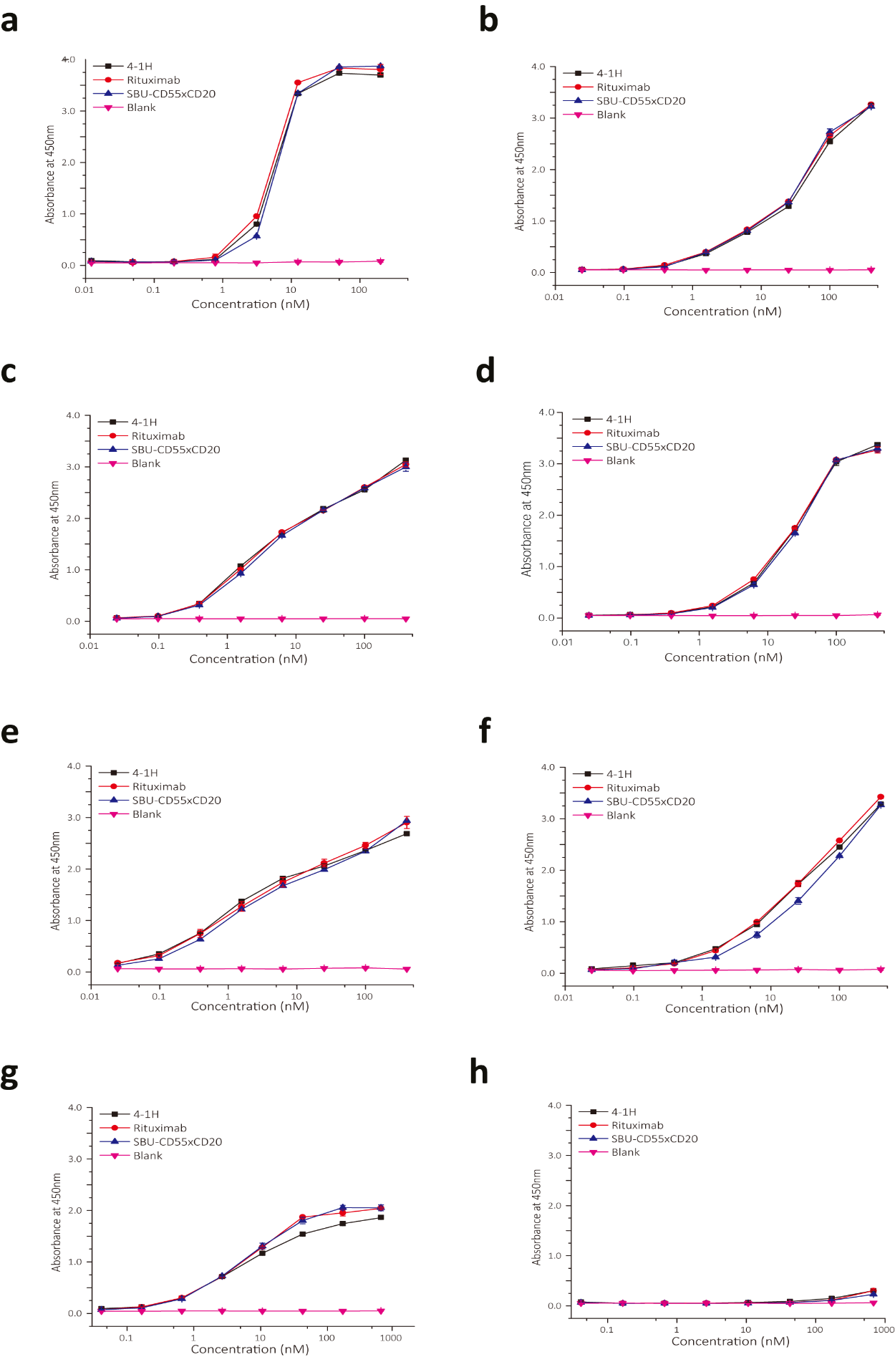
**

**Figure S8.**


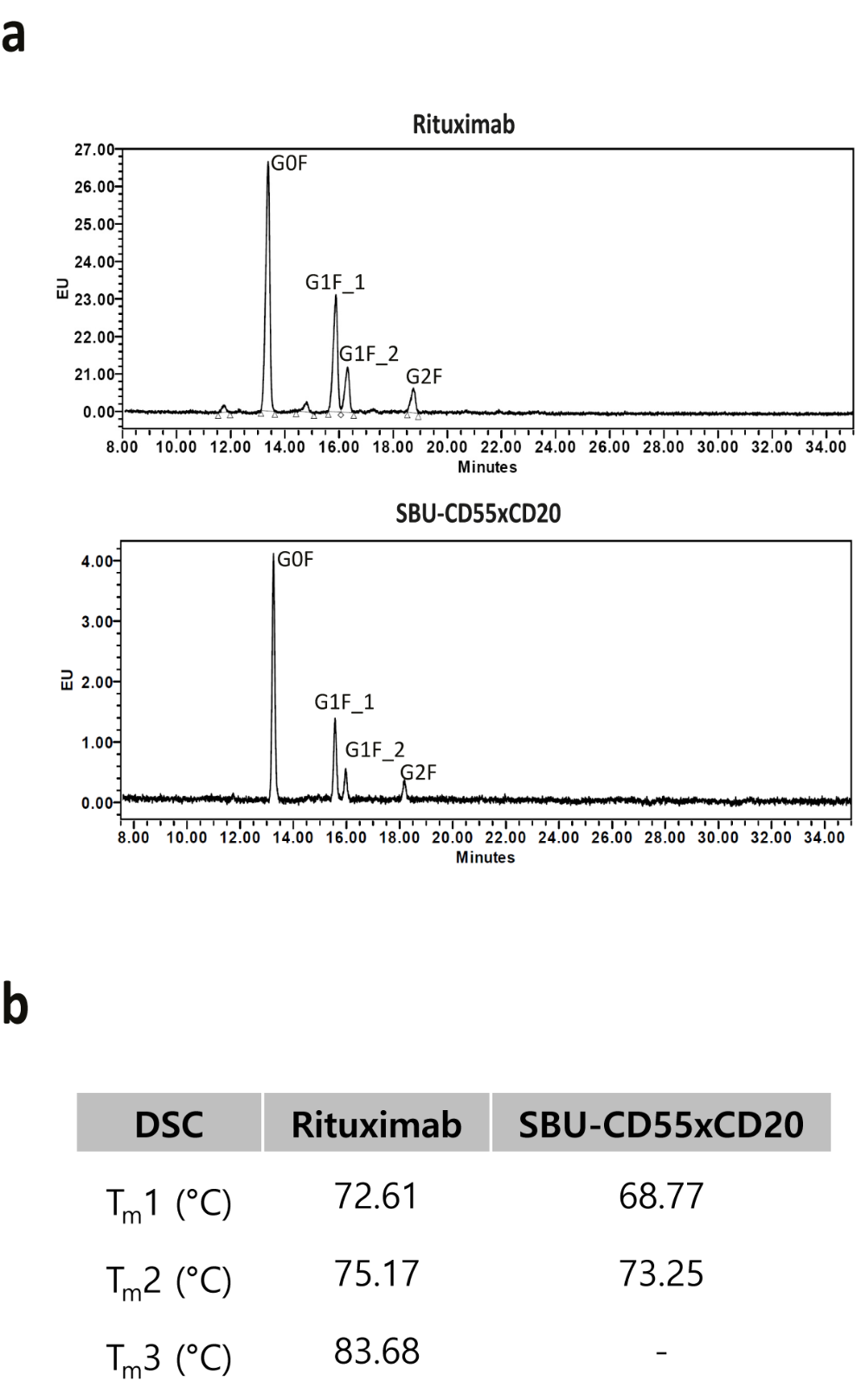


**Figure S9.**


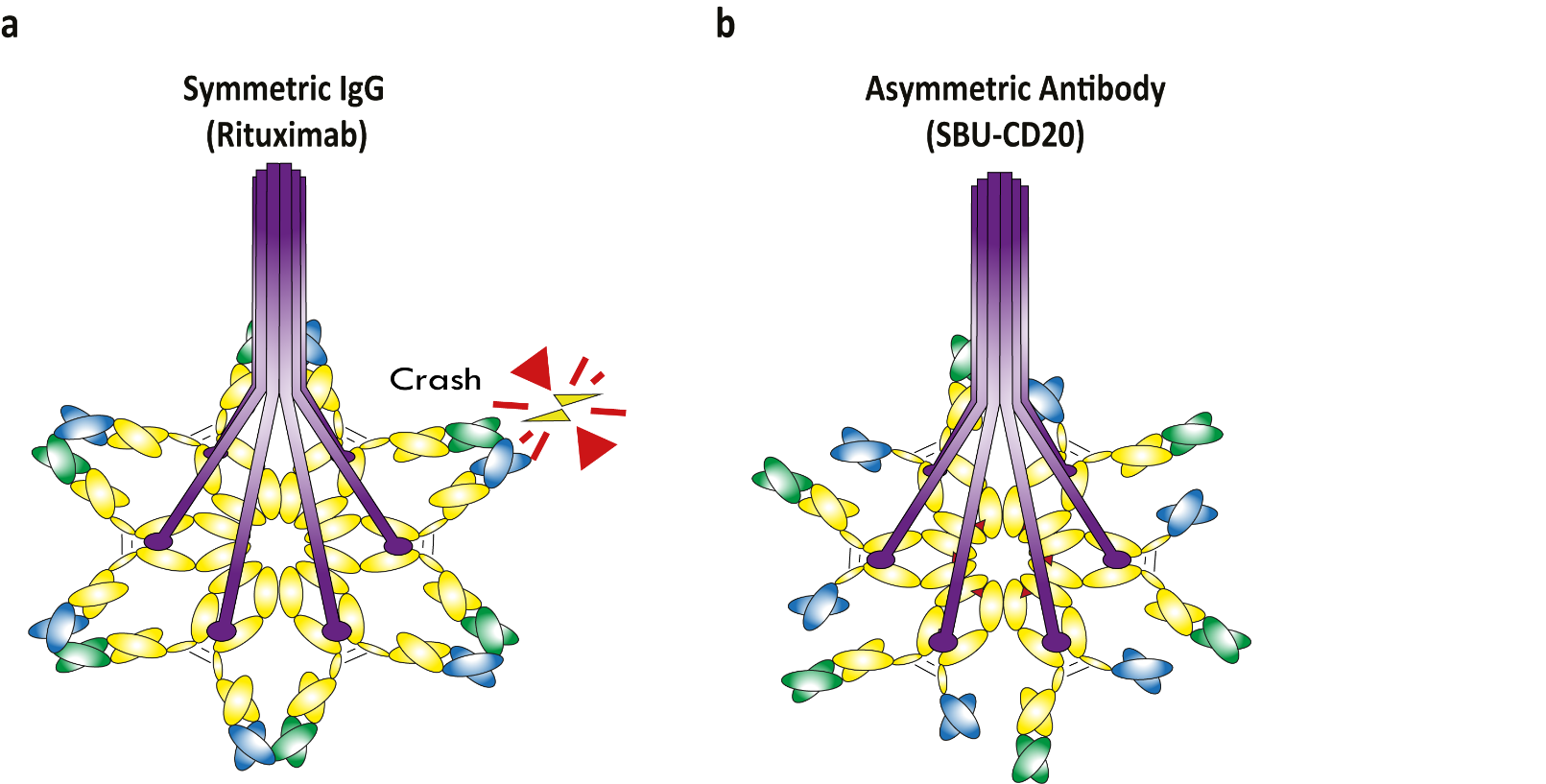


**Figure S10.**


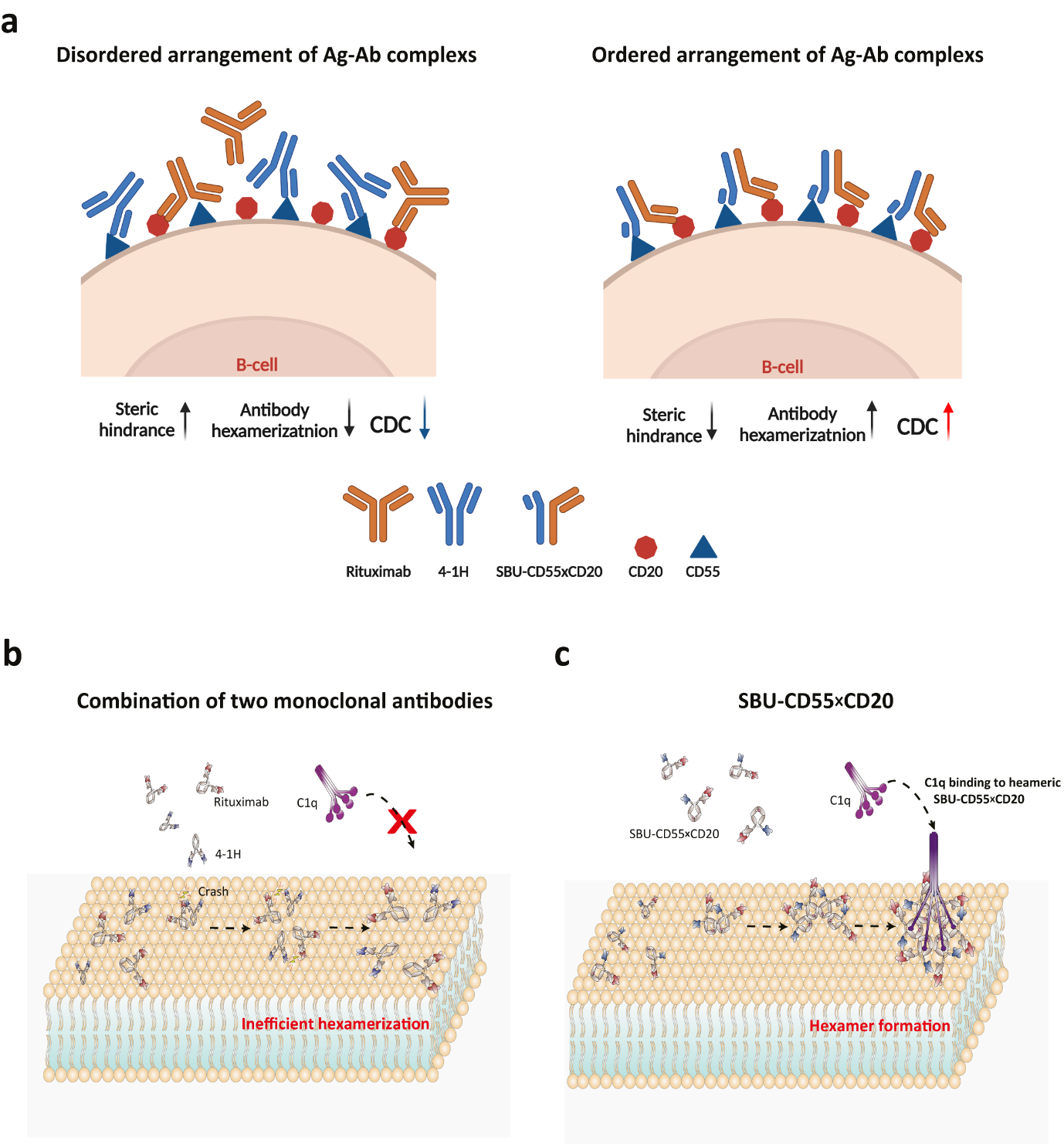


**Figure S11.**

**
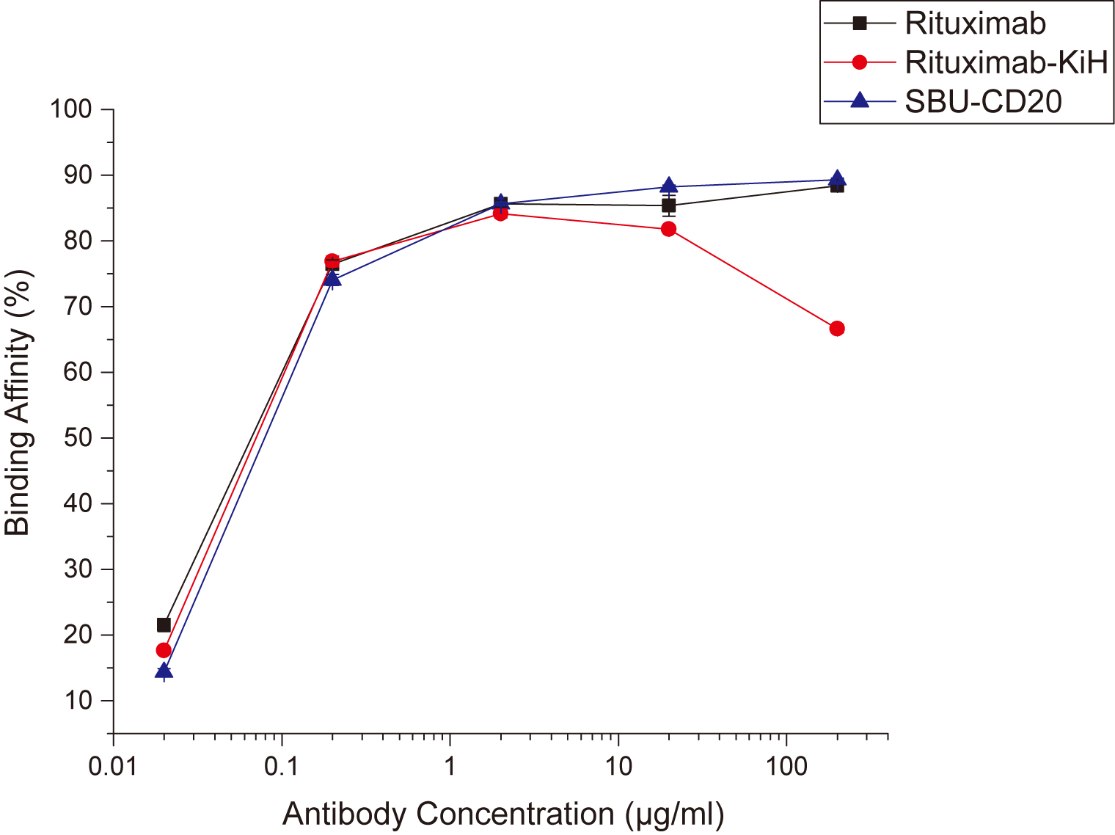
**

**Figure S12.**

**
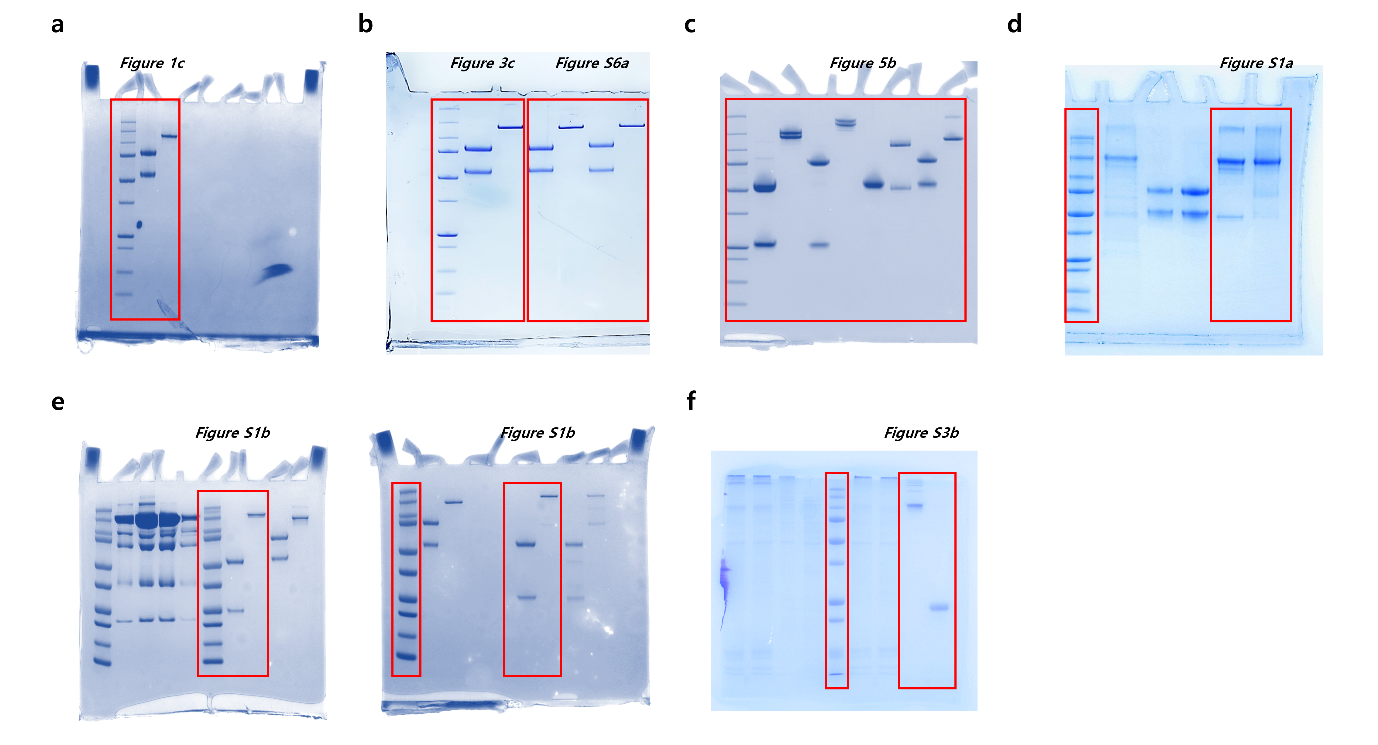
**
